# Forecasting low flow conditions months in advance through teleconnection patterns, with a special focus on summer 2018

**DOI:** 10.1038/s41598-020-70060-8

**Published:** 2020-08-06

**Authors:** M. Ionita, V. Nagavciuc

**Affiliations:** 1 Alfred Wegener Institute Helmholtz Centre for Polar and Marine Research, Bussestrasse 24, 27570 Bremerhaven, Germany; 2 Faculty of Forestry, Ştefan Cel Mare University, 720229 Suceava, Romania

**Keywords:** Climate sciences, Hydrology

## Abstract

Over the past decades, Europe has been affected by several low flow periods which had substantial impacts on the hydrology of the rivers themselves as well as on the society and economy. Low flow periods have a direct impact on the environment, on the inland waterway navigation, on the hydropower production as well as on the sediment management, among others. Similar to floods, low flows are naturally occurring phenomena which can significantly hinder different uses and functions of the rivers and impact the aquatic system and the water quality. Moreover, it is projected that, in the future, climate change might lead to drier summers over the European region and therefore to more frequent and severe low flow periods. The results presented here show that the summer 2018 low flow situation, over the Rhine and Elbe Rivers basin, could have been predicted up to two seasons ahead by using previous months' sea surface temperature, sea level pressure, precipitation, mean air temperature and soil moisture. The lagged relationship between the predictand (e.g. seasonal streamflow) and the climate and oceanic predictors varies between 1 month (e.g. precipitation) up to 6 months (e.g. sea surface temperature). Taking into account that all predictors are available in real-time, the forecast scheme can be used to provide early warnings for the upcoming low flow situations, thus offering the possibility for better management of the water resources.

## Introduction

Drought is among the costliest and damaging disasters in the world and is a naturally occurring phenomenon that can affect large land areas and can prevail over extended periods (e.g. several months up to a few years). Over the last two decades, the European region has witnessed a series of long-lasting dry and hot summers (2003, 2010, 2015, and 2018, among others)^1–3^. For example, the year 2018 over the central part of Europe, especially Germany, was extraordinarily hot and dry. For Germany, the period April–July 2018 was the warmest since 1,880 and different meteorological stations have reached all-time maximum temperature records. This situation was also exacerbated by a rainfall deficit since February 2018. Overall, in Europe, the monetary losses caused by hydrological and meteorological extremes, over the period 1980–2017, amounted to ~ 453 billion Euro^4^. Prolonged dry periods, such as the ones observed in the last decades (e.g. 2003, 2015, and 2018) have emphasized the degree of vulnerability of society to this natural hazard and alerted different governmental agencies and stakeholders regarding the damaging effects drought can have on the economy and society^5,6^. Moreover, the IPCC fifth assessment report^7^ concludes that the magnitude and frequency of extreme events (e.g. droughts, floods, heatwaves, compound events) will increase globally in the future and large areas of the European continent will be exposed to increased drought risk and possibly to more frequent and long-lasting low flow periods. Prolonged low flow periods may result in several types of issues for the society and economy like hindering the inland waterway navigation, lack of drinking water and deterioration of the water quality, reduced irrigation for agricultural purposes, and reduced hydropower production, among others.

River networks provide an important means of transportation both for economical as well as for leisure purposes. Thus, low flow information is often required for a wide range of applications that are most of the time controlled by national and international agencies. For example, inland waterway navigation is often hindered during low flow conditions because the low water levels cannot accommodate ships and vessels anymore and the available water is insufficient for navigational purposes. Since the inland waterway navigation is most of the time dependent on long-term investments and long-term management and the related infrastructure and money cannot be easily relocated, it is imperative to forecast low flows so that the shipping companies are aware of navigation restrictions and have the opportunity to provide alternative means of transport. Currently, there are a large number of drought warning systems available at a global scale^8^^,^ but these systems are providing monthly/seasonal forecasts based either on meteorological drought indices (e.g. Standardized Precipitation and Evapotranspiration Index and/or Palmer Drought Severity Index) or agricultural drought-related indices (e.g. forecast based on soil moisture conditions). European-wide, there is one large operational hydrological forecasting product—the European Flood Awareness System (EFAS). EFAS provides every month an outlook of probabilities of low and high flows for the upcoming 8 weeks (www.efas.eu). Although the EFAS seasonal outlook did manage to predict the summer 2018 low flow conditions up to six weeks ahead in parts of Europe, the forecast signal was rather weak in some areas, especially over western Europe, including Rhine and Elbe rivers (https://hepex.irstea.fr/summer-2018-in-europe/).

Overall, at the European level, there is a lack of a systematic monthly and/or seasonal forecast system with a focus on low flow conditions. One reason for this might be that hydrological data are difficult to be obtained in real-time and the hydrological forecast (e.g. streamflow and water levels) is mainly carried out by national hydrological services, which focus mostly on flood forecasting and to a lesser extent on low flows.

In Germany, almost 6% of the total transported goods are transported per inland waterway vessel (as of 2017). A large part of this happens at around 78% via road traffic, while railway and maritime transport account for around 9% and 7%, respectively. However, for individual goods divisions such as coal, oil and natural gas, coking, and petroleum and chemical products, inland waterway transport is responsible for 10% to 30% of the transport volume and is thus of significantly greater importance^9^. Any slowdown in the navigation time leads to production hindrances in downstream production stages. Inland navigation is also important for foreign trade. In 2017, 23% of the transported volumes were intended for export, 46% came from the import. Facing these issues, it is crucial for low flow management, economy, and society, that more accurate monthly, seasonal and long-term (decades) predictions of low flows become available.

**Figure 1:**
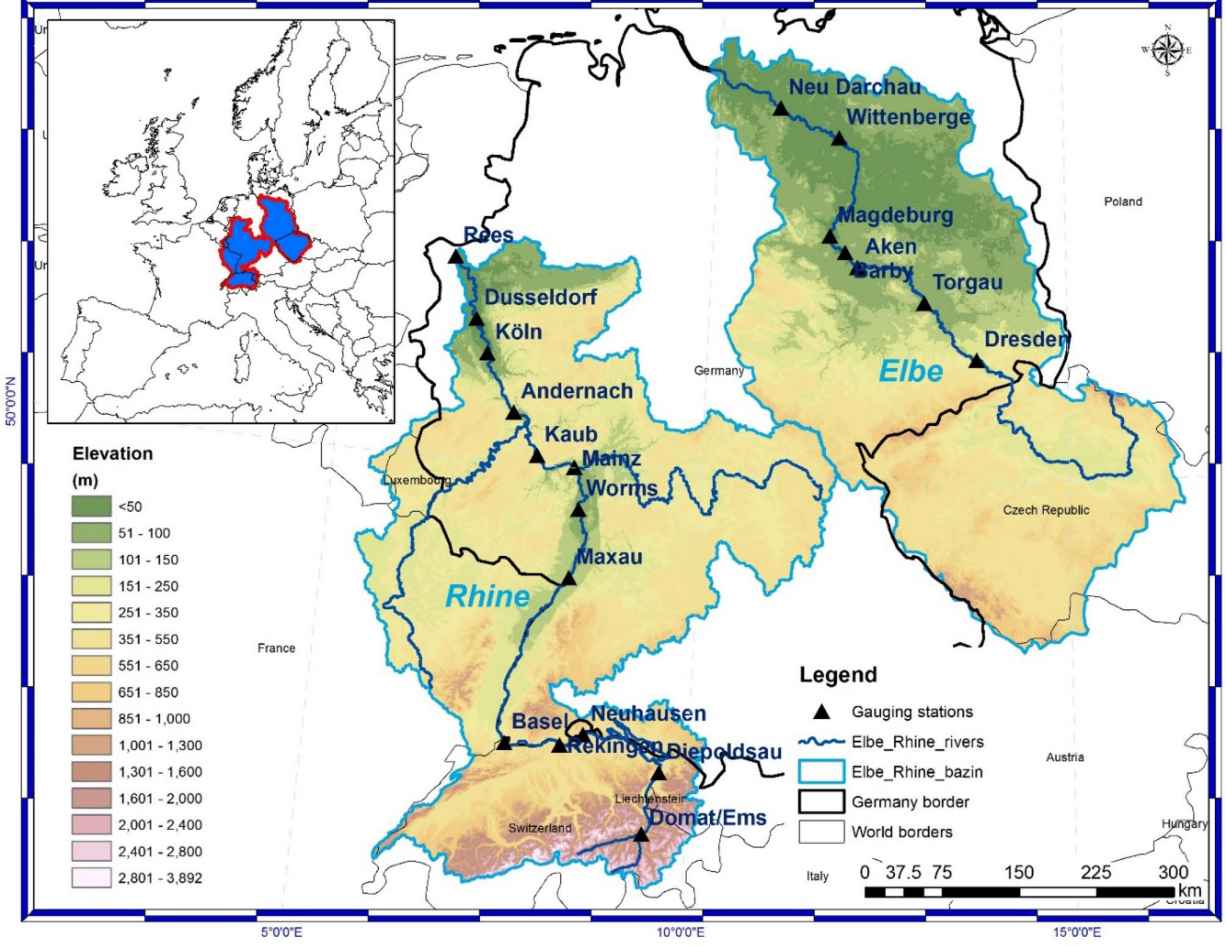
Location and elevation of the two analyzed basins: Rhine (left) and Elbe (right).

Globally, different recognized teleconnection indices like the North Atlantic Oscillation (NAO), El Niño–Southern Oscillation (ENSO), and/or the Pacific Decadal Oscillation (PDO), among others, are used in streamflow forecasting. These indices can provide sources of predictability for streamflow forecasting, over different regions of the world^10–13^. However, these forecasts are not without limitations, because these pre-defined teleconnection indices have a defined spatial scale and use sea level pressure or sea surface temperature data aggregated over specific regions. For example, over Europe, the predictability of streamflow using NAO and ENSO as potential predictors were found to be limited due to non-stationarity^14,15^. One way to overcome the issues of non-stationarity, thus to improve the monthly and seasonal streamflow forecast would be to identify stable predictors, e.g. the relationship between the streamflow and the potential predictors does not change in time^16–18^. This study builds upon an already tested methodology, but with the aim of showing the importance of real-time forecast for low flow periods. For this, we have correlated the seasonal streamflow with large-scale predictors (e.g. precipitation, temperature, sea level pressure, sea surface temperature), in a moving window of 31 years. The results of this analysis are depicted in our study as stability maps, highlighting grid-points where the monthly/seasonal streamflow and the large-scale predictors are significantly correlated at 95%, 90%, 85%, and 80% significance level for more than 80% of the 31-year time windows. The 80% and 85% levels are used as “buffer zones” and only grid cells where the correlation is above 90% significance level, are retained for further analysis. The methodology has been already tested for the prediction of the spring streamflow condition for the Elbe river^16^, as well as for the prediction of the September Arctic Sea ice^19^ and in dendroclimatological studies^20,21^. In this respect, this study describes the performance of a statistical model in predicting low flow situations for the major watersheds in Germany: Rhine and Elbe (Fig. 1). The paper has a special focus on summer 2018, characterized by one of the driest years on record over most of the German territory, with significant consequences for the inland waterway transport, economy, and biodiversity on the Rhine and Elbe rivers.

### Large-scale drivers and regional impacts

**Figure 2:**
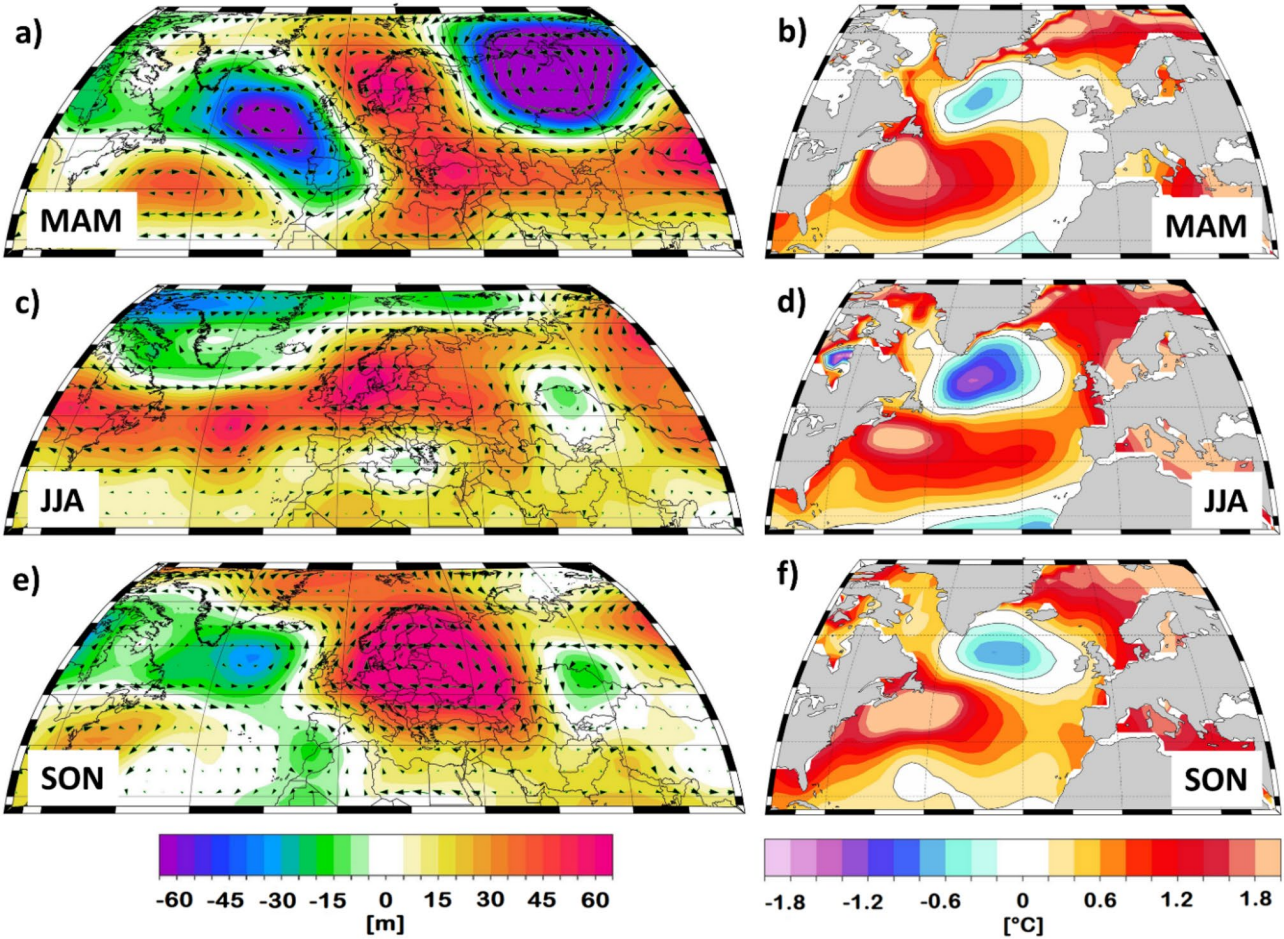
(**a**) Geopotential Height at 500mb (Z500) anomalies averaged over the months March–April–May (MAM) 2018; (**b**) Sea Surface Temperature (SST) anomalies averaged over the months March–April–May (MAM) 2018; (**d**) as in (**a**) but for the months June–July–August (JJA); (**d**) as in (**b**) but for the months June–July–August (JJA); (**e**) as in (**a**) but for the months September–October–November (SON) 2018 and (**f**) as in (**b**) but for the months September–October–November (SON). The anomalies for Z500 and SST are computed relative to the period 1971–2000. Zonal and meridional wind at 500 mb level are added to indicate wind directions.

Throughout the spring, summer, and autumn of 2018, the prevailing large‐scale atmospheric circulation was characterized by positive geopotential height anomalies extending over large parts of the European continent. Spring 2018 (Fig. 2a) was characterized by a Rossby-wave guide with positive geopotential height anomalies over the Fennoscandia extending until the south-eastern part of Europe, one center of negative geopotential height anomalies over the central North Atlantic Ocean and one center of negative geopotential height anomalies over Siberia. The SST anomalies in the North Atlantic Ocean were characterized by a typical tripole-like pattern (Fig. 2b): positive SST anomalies in the central Atlantic Ocean and the Mediterranean Sea; negative SST anomalies south of Greenland and positive SST anomalies poleward of 65°N. This tripole-like pattern is, in general, associated with the occurrence of summer droughts and heatwaves over the central part of Europe^1,22^. Summer 2018 (Fig. 2c) was characterized by positive geopotential height anomalies extending from the central North Atlantic basin until the eastern part of Europe. The tripole-like SST anomalies in the North Atlantic realm persisted throughout the summer months, but with higher amplitudes, especially for the negative SST anomalies southeast of Greenland (Fig. 2d). Throughout autumn 2018 (Fig. 2e) a long-lasting atmospheric blocking situation prevailed over the Scandinavian Peninsula and the central part of Europe. In autumn, the warmth in the Atlantic Basin in the 20–40°N band and north of 65°N and the cooling south of Greenland persisted (Fig. 2f). The tripole-like SST anomalies in the Atlantic Ocean basin, and the prevailing high-pressure systems over most of the European region, which was present throughout the spring, summer and autumn months, suggests that the interplay between the ocean and the atmosphere, associated with the northward shift of the subtropical high, plays an important role in the occurrence of droughts and heatwaves over Europe^1,23,24^. Overall, the complex evolution of prevailing large-scale atmospheric circulation (e.g. long-lasting blocking situations) could, at least partially, explain the exceptionally high temperatures from April to October, especially over Germany. The frequency, magnitude and persistence of atmospheric blocking like conditions is considered to be one of the main important drivers of the large‐scale heat waves and droughts over the European continent^25,26^. A similar situation (e.g. long-lasting blocking situation and anomalous SSTs in the Atlantic basin and the Mediterranean Sea) was observed throughout the hot and dry summer of 2015^1,27^.

**Figure 3:**
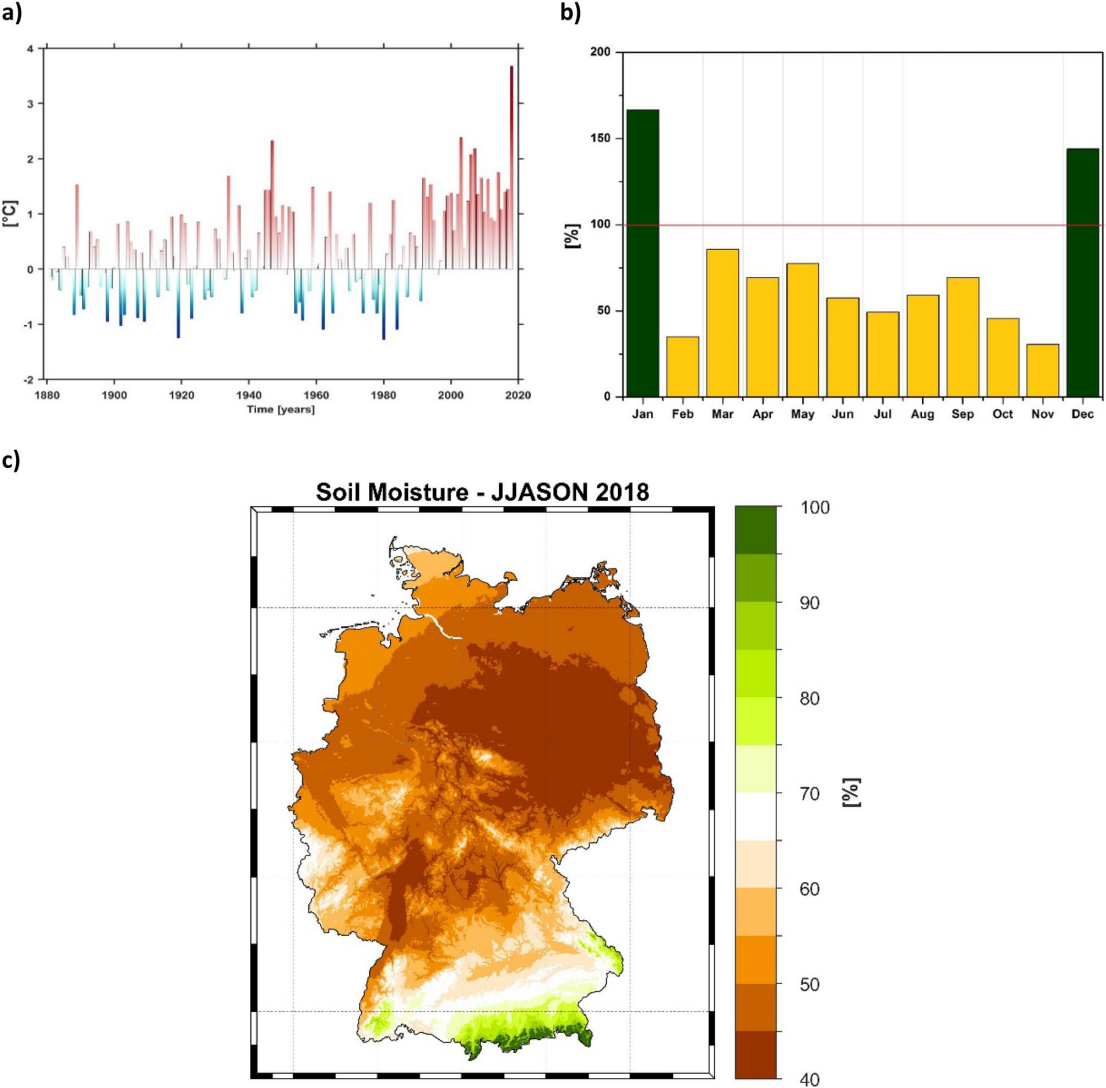
(**a**) Air temperature anomaly averaged over the months April–May–June–July (AMJJ) over the period 1,880–2018. AMJJ 2018 ranks as the hottest year on record; (**b**) Percentage of the monthly precipitation for the year 2018 for Germany, relative to the climatology over the period 1971–2000 and (**d**) June–July–August–September–October (JJASON) soil moisture for 2018. Data source: https://opendata.dwd.de/climate_environment/CDC/.

At the regional scale, the period from April to July 2018 was the warmest in Germany since 1,880′s, when the observational records started (Fig. 3a). Throughout these 4 months, the mean air temperature was more than 1.7 °C above the climatological mean, for all German federal states. Besides, the situation was aggravated by a rainfall deficit from February 2018 until November 2018 (Fig. 3b). Altogether, the Germany-wide average precipitation over the period June to November 2018 period was less than 50% of the usual amount of rain. The most affected areas were the ones in the vicinity of Elbe’s River basin. The reduced precipitation amount led to very small values of the soil moisture over the same period. For example, in the Elbe River basin, the average soil moisture recorded between June and November 2018 was lower than 50% (Fig. 3c).

### Hydrological perspective

From a hydrological point of view, the year 2018 was also exceptional, with negative consequences over different economical and societal sectors, with a special focus on the central and western parts of Europe. According to the European Drought Observatory^28^, much of central and northern Europe was affected by drought, resulting in very dry soil and low river, groundwater and reservoir levels. A remarkable feature over this period was the growth in the spatial extent of low flows across the European river network. The daily streamflow reached minimum values in most central European rivers during late August, persisting until November. Over Germany, long sections of the Rhine, Elbe, Weser, and Main rivers and their tributaries reached the highest hazard class for low flows. Due to the extremely low flow conditions, large chemical companies in Germany had to showdown or radically reduce their production. The impact of the low level on the Rhine river was felt also by the barge market as well as by trading hubs. In August and September 2018, the quantities transported per inland shipping were ~ 20% below the previous year’s level, which also had a negative impact on Germany’s gross domestic product^9^.

**Figure 4:**
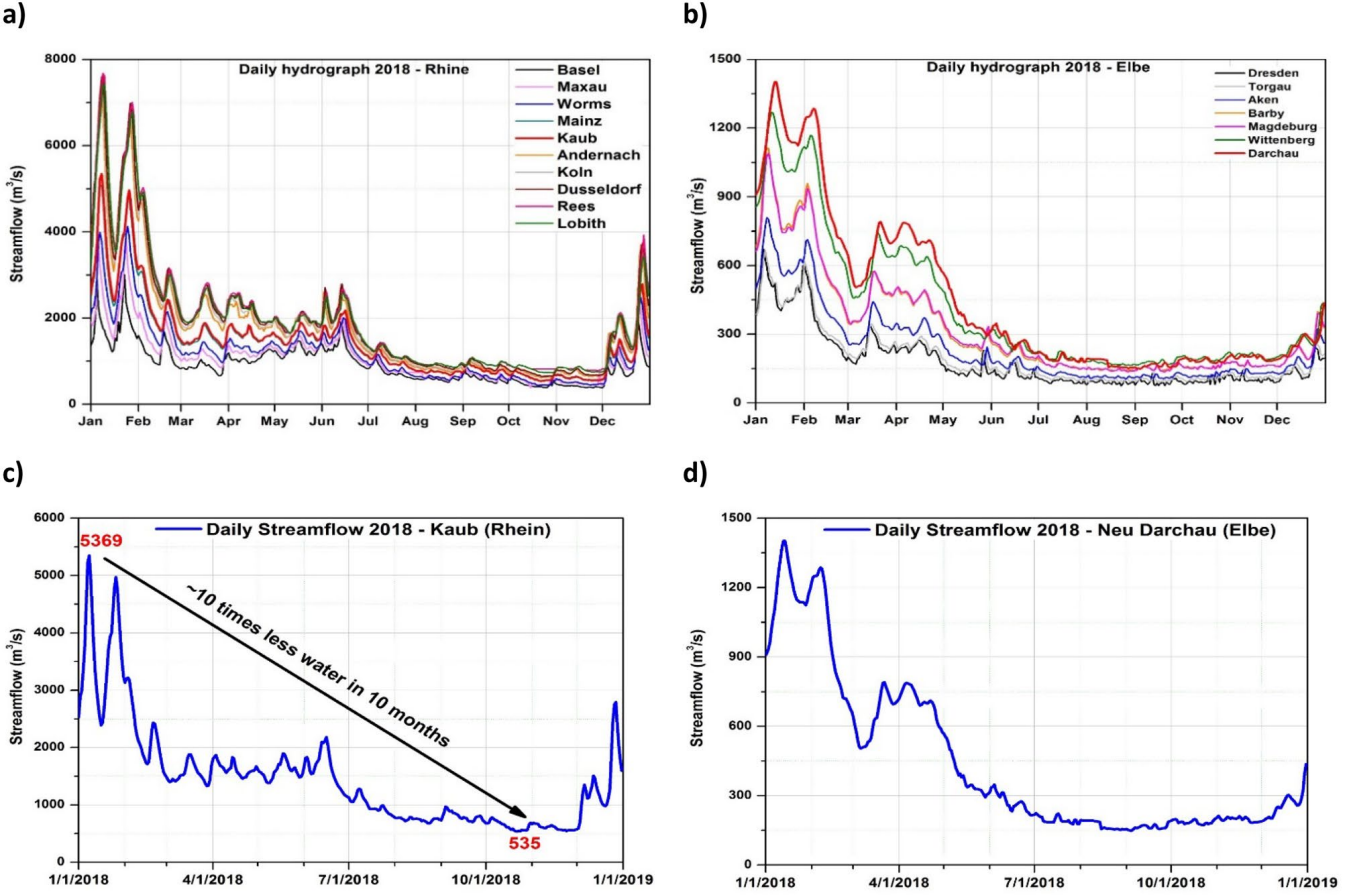
(**a**) The daily hydrograph for 2018 at different gauging stations situated on the Rhine river; (**b**) the daily hydrograph for 2018 at different gauging stations situated on the Elbe river; (**c**) the daily hydrograph for 2018 at Kaub gauging station indicating the abrupt decline in the daily streamflow throughout the year 2018 and (**d**) the daily hydrograph for 2018 at Neu Darchau gauging station.

The daily hydrograph for 2018, at all analyzed gauging stations over the Rhine River basin, shows some interesting features (Fig. 4a). From January to July 2018, there were altering periods of low flows and some high peaks, especially in June 2018. The observed peaks in the Middle and Upper Rhine can be a direct response to the snow and glacier melt over the Alpine regions. Opposite to this situation, from July until the beginning of December 2018 extraordinarily low flow rates have occurred constantly. For example, at Kaub gauging station, at the end of November 2018 the daily streamflow (~ 535 m^3^/s < Q99) was ~ 10 times smaller compared with the daily streamflow at the beginning of January 2018 (~ 5,369 m^3^/s) (Fig. 4c). Over the Elbe River basin, the situation was slightly different in the first 6 months of 2018 (Fig. 4b). The low flow period started at the beginning of June, affecting all the analyzed gauging stations. As in the case of Kaub gauging station, the daily streamflow recorded at the beginning of October 2018 at Neu Darchau gauging station was ~ 10 times smaller compared to the streamflow recorded in January 2018 (Fig. 4d). For the Elbe River basin, the water levels fell below the navigation relevant low level in 90% of the days between June and December 2018^29^.

To test if summer 2018 was a record-breaking one, in terms of hydrological indicators, in this section we analyze the long-term variability of low flows for Rhine River and Elbe River, respectively. The intensity, duration, and frequency of low flow periods vary substantially between regions, seasons, years, and catchment areas. Low flow periods are often defined using a threshold-based methodology, the 30th and 10th percentile of exceedance are most commonly used as a reference threshold^30^. In this study we define low flows as the periods when the observed daily streamflow falls below the threshold defined by the 5th (Q95), 10th (Q90) and 30th (Q70) percentile of the flow duration curve, i.e. the flow exceeded 95%, 90% and 70% of the time. We use Q95 as an indicator for extremely low flow periods, Q90 as an indicator for severe low flow periods and Q70 as an indicator for moderate low flow periods. Table 1 (Rhine) and Table 2 (Elbe) illustrate the range of low-flow thresholds and low-flow conditions for each river and each gauging station. Based on the Q95, Q90, and Q70 we have computed the number of days/year when the daily streamflow was below these thresholds. In Fig. S1, the annual occurrence of the low water classes is shown for Kaub (Fig. S1a) and Köln (Fig. S1b) gauging stations. Significantly low flow events are clearly visible for all gauging stations in the Rhine River basin (not shown), especially in the first half of the twentieth century. The events 1921 and 1949 are visible at all the gauges (Figs. S2–S5). For these two particular years, most of the gauges (Worms, Mainz, Kaub, Andernach, Köln, Dusseldorf, Rees, and Lobith) recorded more than 150 days/year with daily streamflow < Q95. For the 2018 event, the most affected gauges (streamflow values < Q95 for more than 100 days/year) are: Worms, Andernach, Köln, Dusseldorf, Rees, and Lobith. In Fig. S6, the annual occurrence of the low water classes is shown for Neu Darchau (Fig. S6a) and Dresden (Fig. S6b) gauging stations. For the Elbe river, the years 1921, 1947, and 2018 stand out as the most extreme years in terms of low flows. Most of the gauging stations recorded 2018 as the driest year on record, with more than 150 days/year with streamflow < Q95. The most affected gauges are the ones situated downstream Elbe’s River basin (Barby, Neu Darchau, and Magdeburg) (Figs. S7–S8).

**Table 1:** Q70, Q90 and Q95 threshold for the gauging stations located on the Rhine river and the analyzed period.

	Q70 (m^3^/s)	Q90 (m^3^/s)	Q95 (m^3^/s)	Analyzed period
Basel	741	524	451	1869–2018
Maxau	910	667	579	1921–2018
Worms	1,020	739	646	1821–2018
Mainz	1,180	855	752	1931–2018
Kaub	1,190	865	757	1821–2018
Andernach	1,420	1,020	875	1931–2018
Köln	1,490	1,100	950	1817–2018
Rees	1,610	1,200	1,040	1815–2018

**Table 2:** Q70, Q90, and Q95 threshold for the gauging stations located on the Elbe river and the analyzed period.

	Q70 (m^3^/s)	Q90 (m^3^/s)	Q95 (m^3^/s)	Analyzed period
Dresden	184	121	100	1806–2018
Torgau	187	131	114	1935–2018
Aken	243	169	146	1936–2018
Barby	310	218	186	1900–2018
Magdeburg	321	233	199	1931–2018
Wittenberge	407	282	239	1900–2018
Neu Darchau	426	290	247	1875–2018

### Predictability: summer streamflow

The skill of the monthly, seasonal and yearly streamflow forecast is often associated with drivers which represent both slow (e.g. sea surface temperature) and fast varying components (e.g. precipitation, temperature) of the earth system^18^. The slow varying components can be used as potential predictors months up to seasons in advance due to their long-term memory. On shorter time scales (1–3 months), the atmospheric circulation plays also a significant role in the streamflow variability and predictability^31,32^. For each of the 3 months to be forecasted (June/July/August—JJA), a large number of stability maps were produced. The summer streamflow for each gauging station (Kaub and Neu Darchau) was stably correlated with previous months/seasons PP, TT, climate index (CI), SLP, meridional wind at 700 mb (U700), zonal wind at 700 mb (V700), soil moisture (SM) and SST. The time lag used to compute the stability maps varies between 1 up to 9 months for all the analyzed variables.

### Rhine river

**Figure 5:**
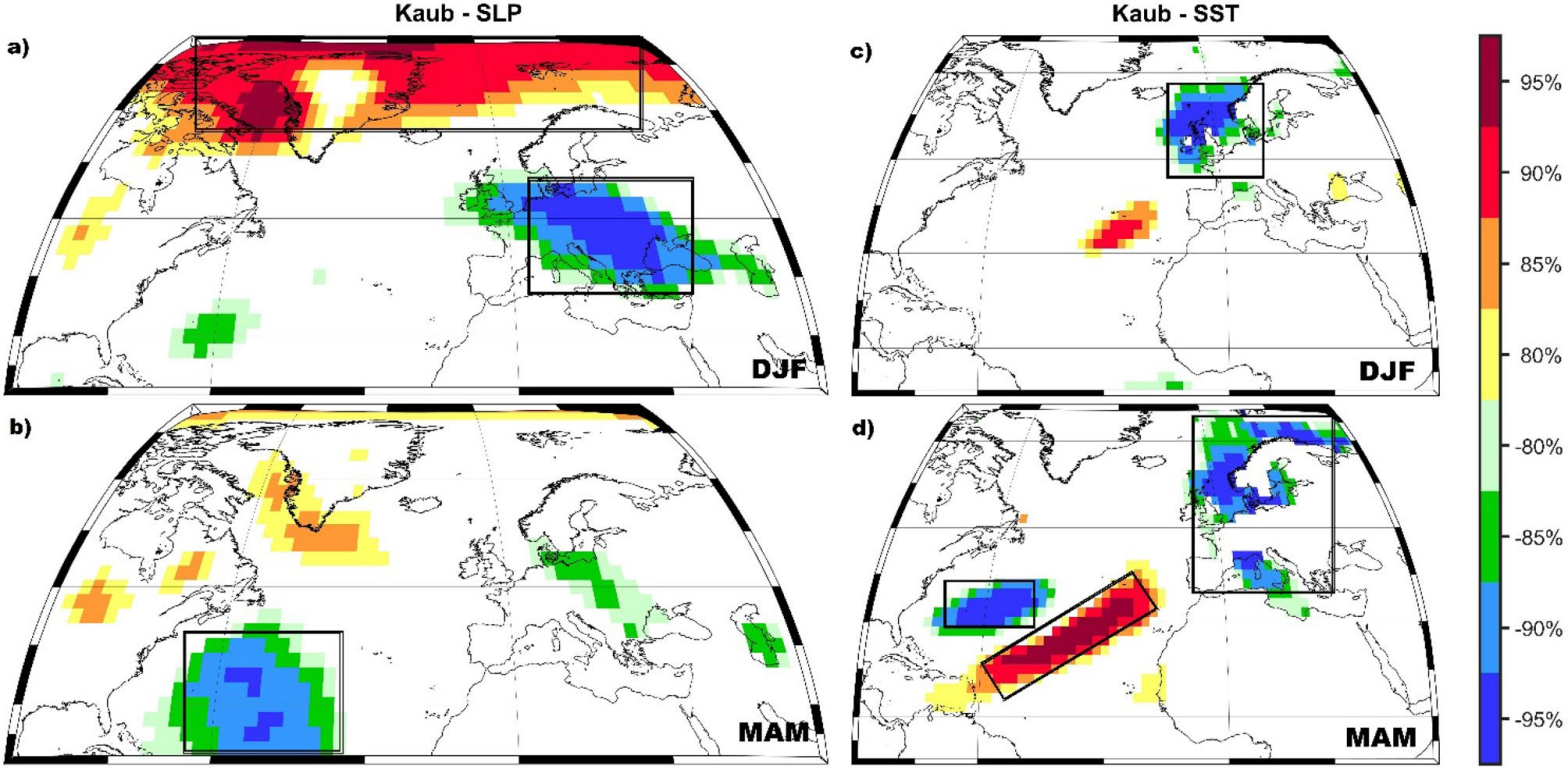
The stability map between the summer streamflow at Kaub gauging station and (**a**) DJF SLP; (**b**) MAM SLP; (**c**) DJF SST and (**d**) MAM SST. The black boxes indicate the regions used for the summer streamflow at Kaub gauging station. Only the regions where the correlation was above 90% significance level were used in the forecast model.

**Figure 6:**
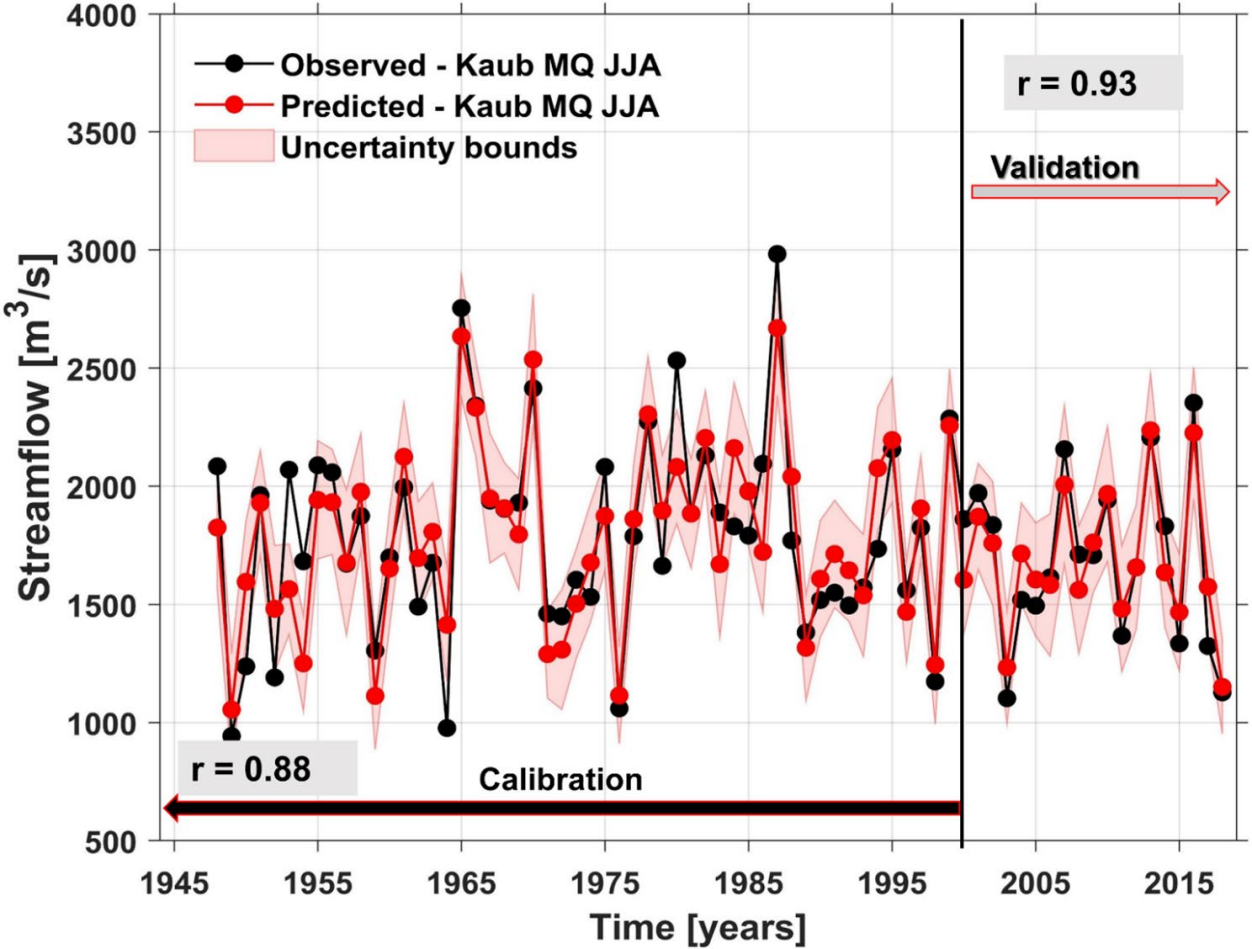
Observed (black) and predicted (red) summer streamflow at Kaub gauging station over the period 1948–2018. The shaded area represents the 95% uncertainty bounds. **r** represents the correlation coefficient for the calibration and the validation period between the observed and predicted summer streamflow.

Based on the stability maps methodology we have identified different predictors with different time lags. For the final forecast model of summer (JJA) streamflow at Kaub gauging station we have extracted all the stable regions shown in the black boxes in Figs. 5 and S9–S10, respectively. Together with these indices identified based on the stability maps, the final forecast model includes also persistence, which is defined as the monthly streamflow from the previous months. The optimal predictors, for the summer Rhine river streamflow, are the regional PP, TT, and CI from the previous seasons (DJF PP, MAM PP, DJF TT, and MAM CI, Fig. S9; previous winter (DJF) and spring (MAM) SLP (Fig. 5a,b); previous winter and spring zonal and meridional wind at 700mb level (Fig. S10), as well as previous winter and spring SST (Fig. 5c,d). The observed and forecasted values of summer streamflow at Kaub gauging station are shown in Fig. 6. As can be inferred from Fig. 6 our statistical approach has a significant high predictive skill and we can properly forecast low flow situations, like the one observed in 2018, at least one season ahead. Over the calibration period (1948–2000) the correlation coefficient between the observed and forecasted streamflow is r = 0.87 (99% significance level), while over the validation period the correlation coefficient between the observed and forecasted streamflow is r = 0.93 (99% significance level). The forecast model for the summer streamflow at Kaub gauging station shows also high predictive skill based on different forecast evaluations metrics: KGE = 0.83 (KGE = 1 indicates a perfect model) and d = 0.93 (d = 1 indicates a perfect match between the forecasted and observed values, d = 0 indicates no agreement at all).

### Elbe river

**Figure 7:**
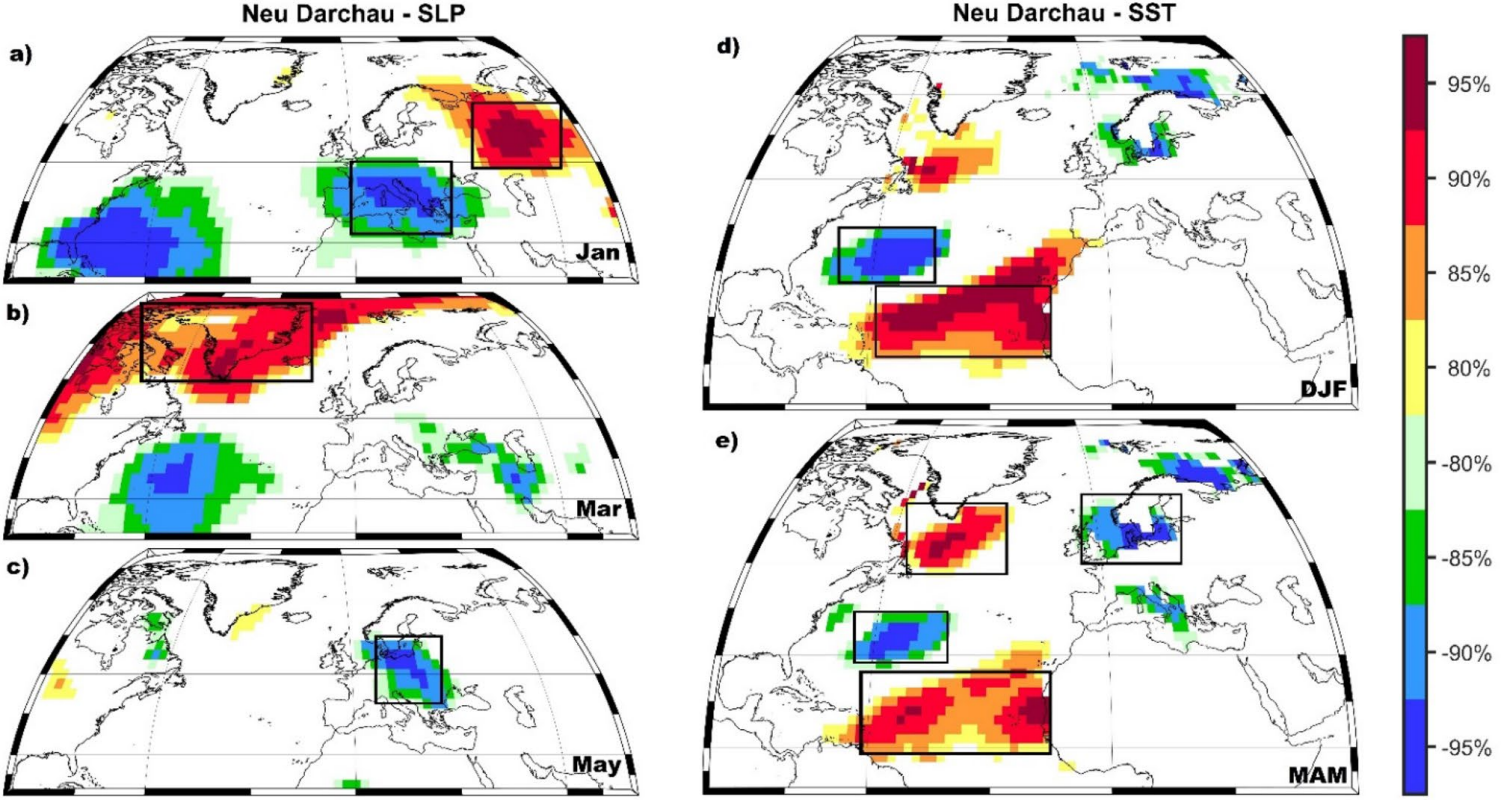
The stability map between the summer streamflow at Neu Darchau gauging station and (**a**) January SLP; (**b**) Mar SLP; (**c**) May SLP; (**d**) DJF SST and (**e**) MAM SST. The black boxes indicate the regions used for the summer streamflow at Neu Darchau gauging station. Only the regions where the correlation was above 90% significance level were used in the forecast model.

**Figure 8:**
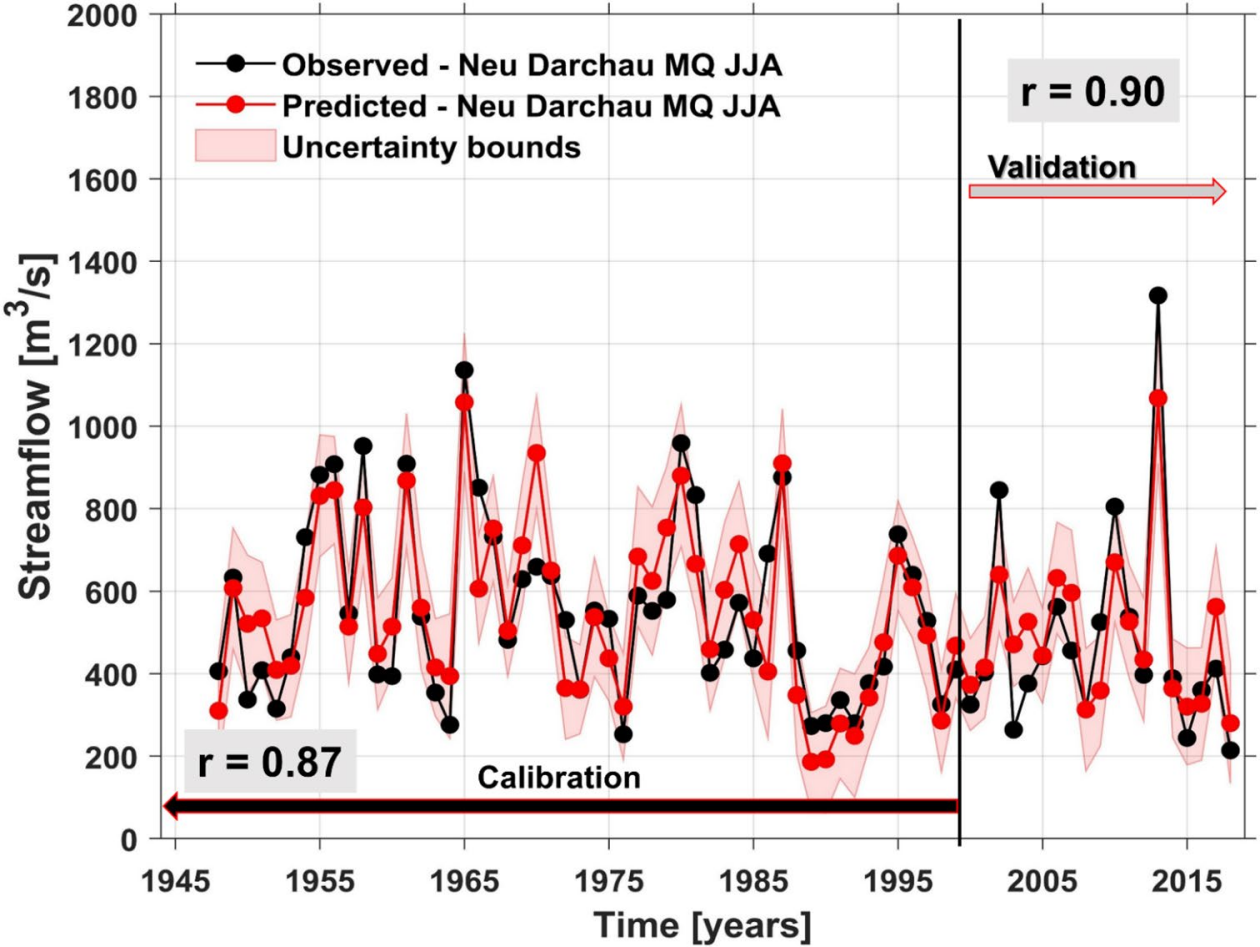
Observed (black) and predicted (red) summer streamflow at Neu Darchau gauging station over the period 1948–2018. The shaded area represents the 95% uncertainty bounds. **r** represents the correlation coefficient for the calibration and the validation period between the observed and predicted summer streamflow.

Following the same steps and methodology as in the case of the Rhine river, we also investigated the skill of our forecast model for the summer streamflow for Elbe river at Neu Darchau gauging station. The stability maps between the summer streamflow at Neu Darchau gauging station and the regional (PP, TT, and CI) and large-scale predictors (SLP, U700, V700, SM, and SST) are shown in Figs. 7 and S11–S12, respectively. For the summer streamflow, at Neu Darchau gauging station the optimal forecast model is based on a combination of May PP, May TT, April and May CI (Fig. S11), January, March and May SLP (Fig. 7a,b,c), May SM and January V700 (Fig. S12) and DJF and MAM SST (Fig. 7d,e). As in the case of the Rhine river, persistence (previous months streamflow) plays also a significant role in Elbe’s summer streamflow forecast. The observed and forecasted summer streamflow at Neu Darchau gauging station is shown in Fig. 8. Over the calibration period (1948–2000) the correlation coefficient between the observed (black line) and forecasted (red line) summer streamflow is r = 0.87 (99% significance level), while over the validation period the correlation coefficient between the observed and forecasted summer streamflow is r = 0.90 (99% significance level). The forecast model for the summer streamflow at Neu Darchau shows also high and significant predictive skill one season ahead (KGE = 0.82, d = 0.93). The low flow conditions in summer 2018 were predictable at least one season ahead, within the 95% uncertainty bounds.

## Discussion and conclusions

In the present study we have investigated the predictability of summer low flow conditions for Rhine and Elbe Rivers, using climatic and oceanic large-scale gridded data from previous months/seasons as potential predictors. The results presented here demonstrate that statistically-based models can skillfully forecast the low flow periods at seasonal time scales if the proper predictors and their location (the so-called stable regions) are properly identified via various statistical methods (e.g. running correlations, stability criteria). In this paper, we focused our analysis on the summer streamflow for two large basins in Europe: Rhine and Elbe Rivers, with a special focus on the summer 2018 low flows. Our results highlight the potential for skillful predictions of summer streamflow, both for the Rhine River as well as for the Elbe River, based on large-scale predictors from slow varying components (e.g. SST) and fast-evolving components (e.g. PP, TT, SLP) of the climate system. In general, large-scale sea surface temperature fluctuations can be linked to different atmospheric circulation patterns which in turn are influencing the hydroclimate variability (e.g. they produce significant precipitation and temperature anomalies), thus affecting implicitly the streamflow variability^25,33–35^. Here we show that summer streamflow is associated with a horseshoe-like SST pattern in the North Atlantic basin in the previous winter and spring. The SST anomalies, identified based on the stability maps (Figs. 5 and 7), are located over regions influenced by different interannual and decadal modes of variability^36^^,^ which affect the prevailing large-scale atmospheric circulation in summer and autumn seasons^32,37^. The summer streamflow of Rhine and Elbe Rivers is stably correlated with the SSTs from large areas in the North Atlantic Basin, which is in agreement with previous studies relating the North Atlantic SST and the climate variability over Europe^16,22,25,26,38,39^. A similar SST pattern has been found to influence the interannual to decadal variability of the Elbe river annual streamflow^31^.

The typical large-scale atmospheric circulation pattern associated with summer low (high) flows at Neu Darchau gauging station, is characterized by a Rossby wave train in the Z500 summer field, featuring a center of positive (negative) Z500 anomalies over the eastern part of Canada, a center of negative (positive) Z500 anomalies over the central North Atlantic basin and a center of positive (negative) Z500 anomalies over the central part of Europe (Fig. S13a—low flows, Fig. S13b—high flows). The pattern observed in the case of low flows (Fig. S13a), especially the high-pressure center over the central part of Europe, is associated with the advection of dry and warm air from the eastern part of Europe over the analyzed region, reduced precipitation, and long-lasting heatwaves^1^. A similar Rossby wave structure, in summer, was observed as a response to persistent SST anomalies in the North Atlantic Ocean (their Fig. 3c)^40^. Gastineau and Frankignoul^40^ have shown that altering SST anomalies in the North Atlantic Ocean, featuring negative (positive) SST anomalies in the Gulf Stream region, positive (negative) SST anomalies on the west coast of Africa, positive (negative) SST anomalies south of Greenland and negative (positive) SST anomalies in the Nordic Seas, precede by ~ 3 months an anticyclonic (cyclonic) circulation over the subpolar North Atlantic and a cyclonic (anticyclonic) circulation over the central part of Europe in summer. The location of the SST anomalies, with altering signs over the whole North Atlantic basin, identified by^40^ is similar to the locations identified as potential predictors in the SST field for Rhine and Elbe. Overall, the response of the summer atmospheric circulation to the horseshoe-like pattern in the SST field results from a combination of both subpolar and tropical forcing^40^.

The lag between the atmosphere and the ocean can be a consequence of the evolution of the jet stream^41^^,^ especially on seasonal time scales. In general, the maximum amplitude of the jet stream is located in the near vicinity of the largest baroclinicity, where large temperature gradients between the ocean and land are observed^42^. In winter the jet stream maximum is situated over the east coast of the U.S., while in spring and summer the jet moves north-eastward due to the warming of the North American continent and the poleward shift of the descending branch of Hadley Cell^42,43^. In their paper, Ossó et al. (2018)^41^ have shown that the summer weather over the western part of Europe is predictable from the late winter and spring SST from the North Atlantic basin and they suggest that the summer atmospheric circulation over the western part of Europe represents the surface response of a poleward displacement of the North Atlantic jet stream. Thus, we argue that persistent winter and spring SSTs act as a precursor for the prevailing large-scale circulation in summer and the location of the storm tracks, which in turn affects the hydroclimate over the European region, including the frequency of low and high flows.

The relationship with PP, TT, and CI are restricted, in the current study, just to regional scale, due to the data availability for a near real-time forecast. In a previous study, Ionita et al.^16^ have shown that Elbe spring streamflow is also stably correlated with PP and TT both at a regional scale as well as at a hemispheric scale. The summer streamflow at Kaub shows also stable and significant correlations with previous winter SLP over the Arctic basin and over the central part of Europe and previous spring SLP anomalies over the Gulf of Mexico. The stability map between summer streamflow at Kaub and winter SLP projects onto the negative phase of the Arctic Oscillation^44^. The AO can generate also the tripole-like SST anomalies in the North Atlantic basin, associated with the predictability of the summer streamflow, mainly through changes in the turbulent energy flux^36,45,46^. This in agreement with the horseshoe-like pattern in the SST field, identified based on the winter and spring stability maps and used as predictors in our statistical model, which resembles the one associated with the negative phase of the AO/NAO in winter and spring^47^. The SLP anomalies associated with the summer streamflow at Neu Darchau are located over the central part of Europe and Siberia. The SLP anomalies over the central part of Europe can trigger enhanced precipitation or snow cover in winter, thus high streamflow and saturated soil moisture over the basin area. This could have significant implications for the summer streamflow via the soil moisture feedback.

Our results show that previous winter and spring regional and large-scale climatic and oceanic variables provide a significant source of predictability for the summer streamflow, especially for low flow years (e.g. 2015, 2018). There are also extreme cases (e.g. 2003) when the observed streamflow at Neu Darchau falls outside the uncertainty bounds of our forecasted streamflow. This might be due to the fact that, for example, summer 2003 was the hottest summer over the last 500 years^48^ and our statistical model was not able to fully capture the influence of the extreme magnitude of the temperature and soil moisture anomalies on the summer streamflow prediction. Overall, the summer streamflow anomalies for Rhine and Elbe are related not only with regional winter and spring climatic anomalies (e.g. PP, TT, CI, and SM) but also with climatic and oceanic anomalies from several key regions located far from Rhine and Elbe River basins. Previous studies have shown that winter and spring streamflow at the European scale have a much higher predictive skill compared to the summer streamflow^15^. Here we show that by using the proper predictors from key regions, the summer streamflow for two major European rivers has a high predictive skill, which can provide valuable guidance for the water management in the Rhine and Elbe basin areas, with significant consequences for the society and economy.

## Data and methods

### Data

The main quantity analyzed in the current study is the streamflow data measured at Kaub gauging station (Rhine River) and Neu Darchau gauging station (Elbe River) (Fig. 1). The streamflow data has been provided by the Global Runoff Center (GRDC) in Koblenz. For the study presented here, the following data gridded datasets are used: precipitation (PP) and temperature (TT) data, at country level, available from the Deutscher Wetterdienst FTP server (ftp://opendata.dwd.de/climate_environment/CDC/grids_germany/monthly/), with a 0.1° × 0.1° spatial resolution and covering the period from January 1948 up to present. Based on the PP and TT datasets we have defined also a climate index (CI) which is computed by subtracting the standardized temperature from the standardized precipitation. Negative values of CI are an indicator of dry and warm conditions, whereas positive values of CI are an indicator for wet and cold conditions. The Soil Moisture (SM), soil temperature in the first 10 m (TT10), meridional wind at 700mb (U700), zonal wind at 700mb (V700) and the sea level pressure (SLP) data sets are provided by the National Centre for Atmospheric Research (NCAR), they cover the period from January 1948 to present and have a spatial resolution of 2.5° × 2.5°^49^. For the global sea surface temperature, we use the Extended Reconstructed Sea Surface Temperature (ERSST.v5)^50^ dataset, which covers the period January 1948—present and it has a spatial resolution of 2° × 2°. All the aforementioned datasets are updated in real-time, thus allowing a forecast to be issued at the beginning of each month.

### Methodology

For the analysis and the forecast model, all data sets were separated into two parts: the calibration period (1948–2000) and the validation period (2000–2018). The final forecast model is obtained by employing stepwise multiple regression analysis^51^ (*see Supplementary file*). The methodology used for the seasonal streamflow forecast is based on a methodology similar to the one used previously for the monthly prediction of Elbe river streamflow and the Arctic Sea Ice^16,19^. A schematic view of the methodology is given in Fig. S14. The basic idea of this procedure is to identify regions with stable teleconnections between the predictors (e.g. PP, TT, CI, SLP, SST) and the predictand (monthly/seasonal streamflow). To obtain the stability maps the seasonal streamflow has been correlated with the potential predictors from previous months/seasons, in 31 years moving window. The correlation is considered to be stable for those grid-points where streamflow and the potential predictors (e.g. SLP, SM, TT10, U700, V700, SST, PP, TT) are significantly correlated at the 80%, 85%, 90% and 95% significance level for more than 80% of the 31-year windows, covering the period 1948–2000. Such maps (e.g. Fig. 7) are referred to in this study as stability maps and the statistical significance of the correlation coefficient is tested using a *Student t* test. For the current study, we took into account just the correlations stable above the 90% significance level.

## Supplementary information

Supplementary Information.

## Data Availability

All datasets used as input in our study can be found in the respective references.All datasets used as input in our study can be found in the respective references.
