# Supplementary material for: Forecasting low flow conditions months in advance through teleconnection patterns, with a special focus on summer 2018
Source: Sci Rep. 2020 Aug 6;10:13258. doi: 10.1038/s41598-020-70060-8 (PMC7411071; doi:10.1038/s41598-020-70060-8)
Supplement: Supplementary file 1 — Supplementary Information. [file 41598_2020_70060_MOESM1_ESM.pdf]

Supplementary file

Forecasting low flow conditions months in advance through teleconnection patterns, with a special focus on summer 2018

M. Ionita[1*], V. Nagavciuc[1,2]
[1]Alfred Wegener Institute Helmholtz Center for Polar and Marine Research, Bremerhaven, 27570, Germany
[2]Faculty of Forestry, Ștefan cel Mare University, Suceava, 720229, Romania

[*]Corresponding author:

Email: Monica.Ionita@awi.de

Address: Alfred Wegener Institute Helmholtz Centre for Polar and Marine Research
Bussestrasse 24
D-27570 Bremerhaven
Telephone: +49(471)4831-1845

Fax: +49(471)4831-1271

1. Study area

Rhine River (*Figure 1*) is one of the largest and most important rivers in Europe. It originates from the southeastern Swiss Alps at an elevation of 2345 m, and it flows through Liechtenstein, Austria, Germany, and France, before draining into the North Sea in the Netherlands. It has a mean streamflow of about 2300 m^3/s and a total length of 1230 km. The river basin covers an area of about 185,260 km^2, being inhabited by about 58 million people. It covers different geographical regions from alpine area upstream to lowlands. The Rhine basin (185,000 km^2) can be divided into the Alpine area upstream from Basel (Switzerland) and the middle and lowland parts, downstream. Downstream from Basel, the Rhine is supplied by several large tributaries, such as the Neckar, the Main and the Mosel (*Figure 1a*). The precipitation ranges from less than 200 mm/year in the central part to 3500 mm/year in the mountain regions[1].

The Elbe River (*Figure 1*) is one of the major rivers of Europe. It originates from the Giant Mountains in the northern Czech Republic at an elevation of 1383 m and flows into the North Sea at Cuxhaven (Germany), with a mean annual streamflow at the mouth of about 861 m^3/s. From a total length of 1094 km, 367 km are located in the Czech Republic and 727 km in Germany, while much smaller parts lie in Austria and Poland. The Elbe basin has a catchment area of 148,268 km^2, which makes it the fourth largest in Europe and covers different geographical regions from middle mountain ranges in the west and south to large lowlands in the central, northern, and eastern parts. The basin is inhabited by 24.5 million people. The Elbe River is located in a transition zone between the maritime and the continental climate, where the temperature indicates strong intra-annual variability, thus influencing the evaporation and it is the driest river in Germany due to low precipitation levels, with an average of about 659 mm/year. The precipitation ranges from less than 450 mm/year in the central part to 1600 mm/year in the mountains[1].

2. Multiple Linear Regression. For the forecast, all data sets were separated into two parts: (1) the calibration period (1948–2000) and (2) the validation period (2000–2018). The optimal predictors are identified by employing stepwise multiple regression analysis[2]. Although the method identifies multiple stable regions for each climate/oceanic parameters, after applying the stepwise multiple regression analysis the optimal model is based just on the most significant regions. The regression equation given by the regression model is:

$$Y = \beta_0 + \beta_1 x_1 + \beta_2 x_2 + \cdots + \beta_n x_n + \varepsilon$$

Where *Y* represents the streamflow data index, β_o, β_1, β_2,…β_n are constants determined by the least squares procedure, x_1, x_2,…x_n the predictors used (e.g. SLP, PP, TT, etc) and ε the error.

In stepwise regression, each predictor is prioritized taking into account its correlation coefficient with the predictand and its added to the model gradually. As the predictors are added, the *F* statistic is used to determine whether or not they are significant for the final regression equation (*F* statistics are set to 0.05 and 0.1, respectively). We use stepwise regression because it has the possibility to prioritize predictors based on the partial correlation, indicating that high and significant correlations reflect underlying physical processes.

3. Skill measures

To better assess the skill of the forecast (*Table S1*), different statistical metrics have been employed: mean absolute error (MAE), mean square error (MSE), root mean square error (RMSE), normalized root mean square error (NMRSE), Nush - Sutcliffe Efficency (NSE), modified NSE (mNSE), relative NSE (rNSE), index of agreement (d), coefficient of persistence (CP) and coefficient of determination (R^2).

1. Mean absolute error (MAE)

$$mae = \frac{1}{N}\sum_{i=1}^{N}|(S_i - O_i)|$$

2. Mean square error (MSE)

$$mse = \frac{1}{N}\sum_{i=1}^{N}(S_i - O_i)^2$$

3. Root mean square error (RMSE)

$$rmse = \sqrt{\frac{1}{N}\sum_{i=1}^{N}(S_i - O_{i)}^2}$$

4. Normalized root mean square error (NRMSE)

$$nrmse = 100\frac{\sqrt{\frac{1}{N}\sum_{i=1}^{N}(S_i - O_i)^2}}{nval}$$

where $$nval = \begin{cases} sd(O_i), & norm = "sd" \\ O_{max} - O_{min}, & norm = "maxim" \end{cases}$$

5. Nush - Sutcliffe Efficiency (NSE)

$$NSE = 1 - \frac{\sum_{i=1}^{N}(S_i - O_i)^2}{\sum_{i=1}^{N}(O_i - \bar{O})^2}$$

NSE (Nush and Sutcliffe, 1970)[3] ranges from -Inf to 1. Essentially, the closer to 1, the more accurate the model is. NSE = 1 indicates a prefect forecast model, NSE = 0 indicates that the model predictions are as accurate as the mean of the observed data and -Inf < NSE < 0, indicates that the observed mean is better predictor than the model.

6. Modified NSE (mNSE)

$$mNSE = 1 - \frac{\sum_{i=1}^{N}|S_i - O_i|^j}{\sum_{i=1}^{N}|O_i - \bar{O}|^j}$$

7. Relative NSE (rNSE)

$$rNSE = 1 - \frac{\sum_{i=1}^{N}(\frac{S_i - O_i}{\bar{O}})^2}{\sum_{i=1}^{N}(\frac{O_i - \bar{O}}{\bar{O}})^2}$$

8. Index of Agreement (0 <= d <= 1)

$$d = 1 - \frac{\sum_{i=1}^{N}(O_i - S_i)^2}{\sum_{i=1}^{N}(|S_i - \bar{O}| + |O_i - \bar{O}|)^2}$$

The Index of Agreement (d) developed by Willmot (1982)[4] as a standardized measure of the degree of model prediction errors and varies between 0 and 1. A value of 1 indicates a perfect match and 0 indicates no agreement at all.

9. Coefficient of persistence (0 <= CP <= 1).

$$CP = 1 - \frac{\sum_{i=2}^{N}(S_i - O_i)^2}{\sum_{i=1}^{N-1}(O_{i+1} - O_i)^2}$$

The coefficient of persistence ranges from 0 to 1, with CP = 1 being the optimal value and it should be larger than 0 to indicate a minimally acceptable performance model.

References

1. Pfeiffer, M. & Ionita**,** M. Assessment of Hydrologic Alterations in Elbe and Rhine Rivers, Germany. *Water* **9(9)**:684 (2017).

2. Von Storch, H. & Zwiers, F.W. Statistical Analysis in Climate Research, Cambridge University Press, Cambridge (1999).

3. Nash, J. E. and Sutcliffe, J. V. 1970: River flow forecasting through conceptual models, Part I - A discussion of principles, J. Hydrol., 10,282–290.

4. Willmot, C. J. (1981) On the validation of models, Physical Geography, 2,184–194.

a)

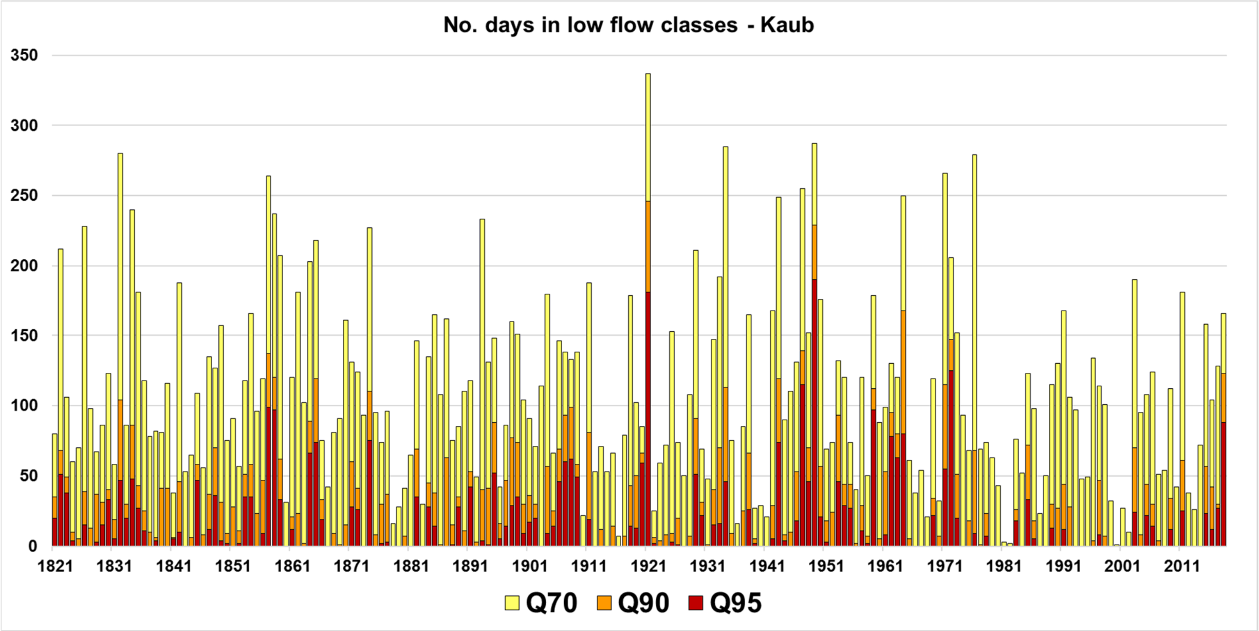

b)

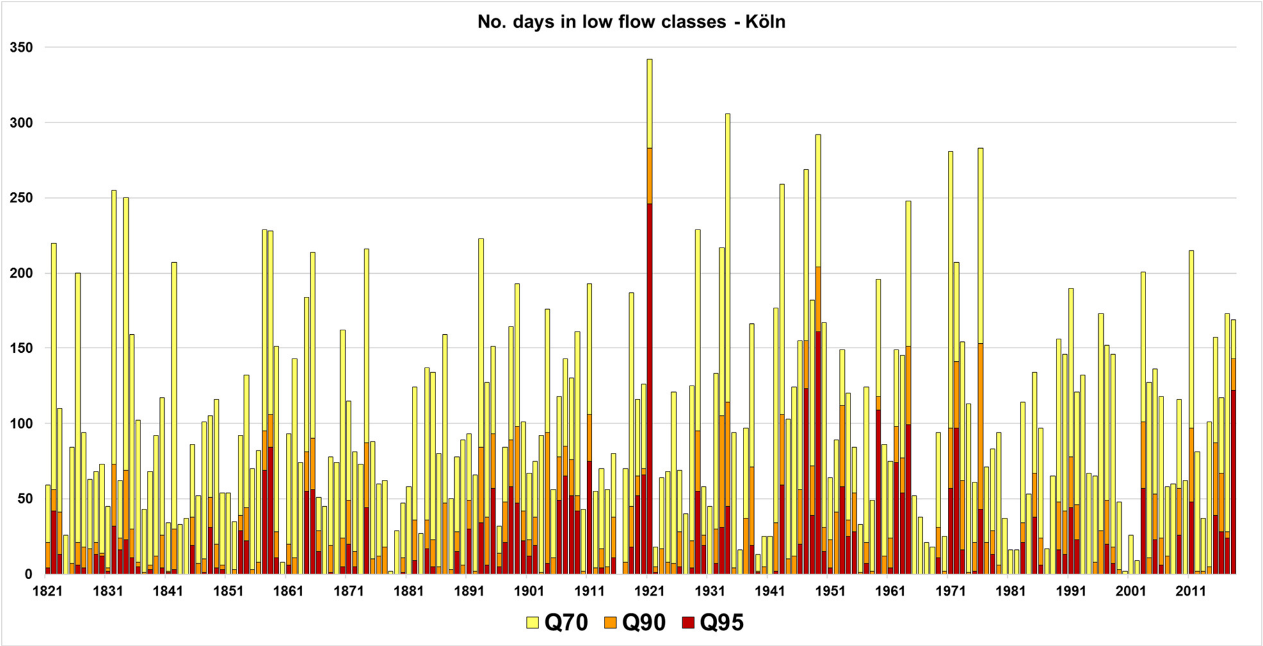

Figure S1. a) Number of days in low flow classes at Kaub gauging station over the period 1821 – 2018 and b) Number of days in low flow classes at Köln gauging station over the period 1821 – 2018

a)

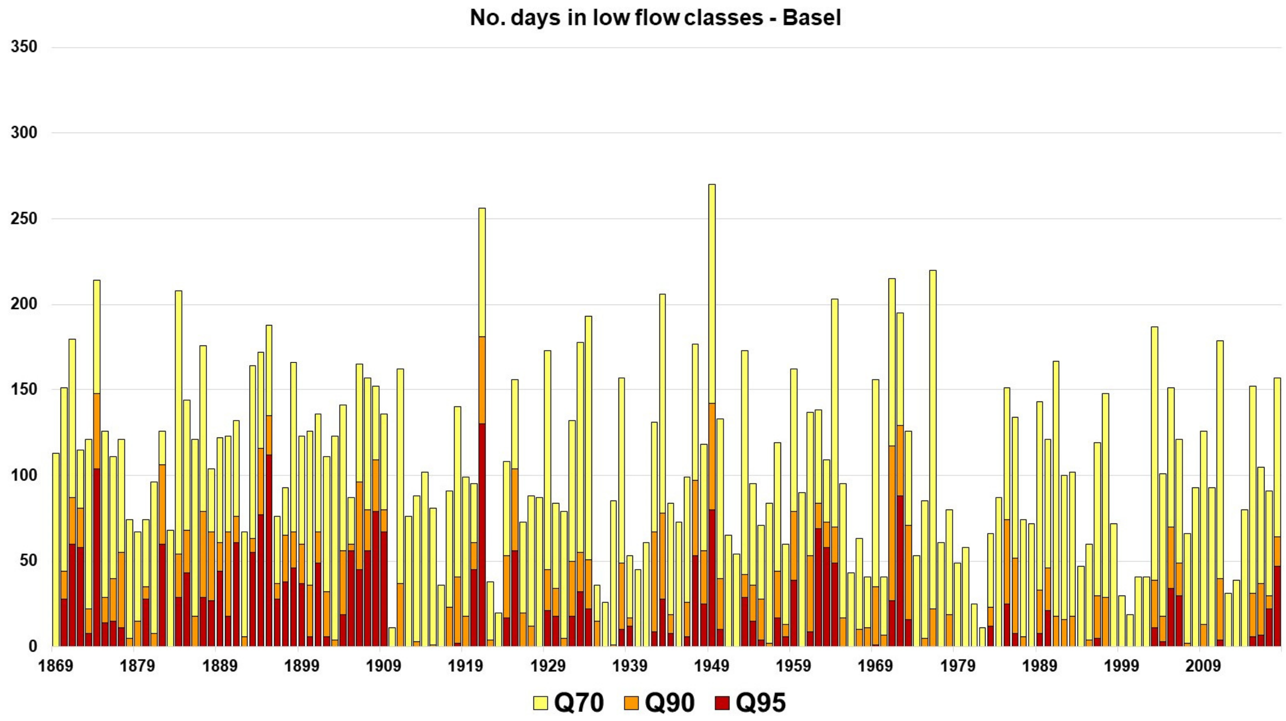

b)

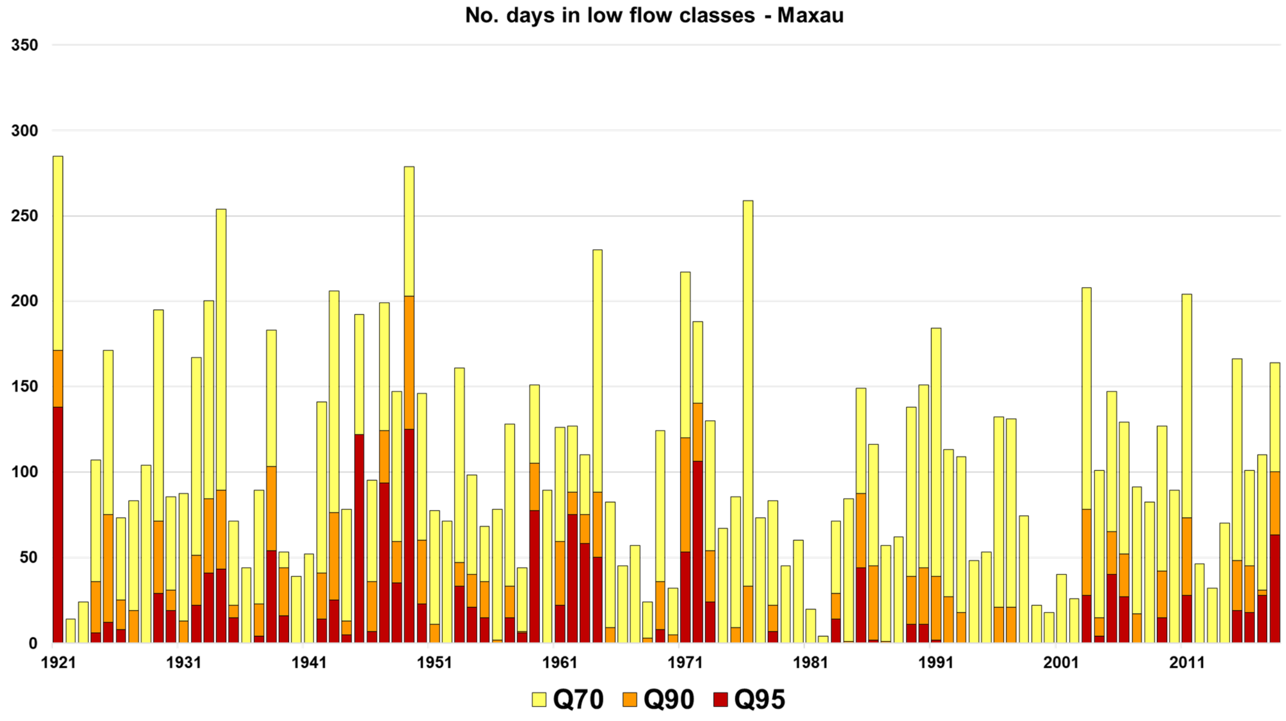

Figure S2. a) Number of days in low flow classes at Basel gauging station over the period 1869 – 2018 and b) Number of days in low flow classes at Maxau gauging station over the period 1921 – 2018

a)

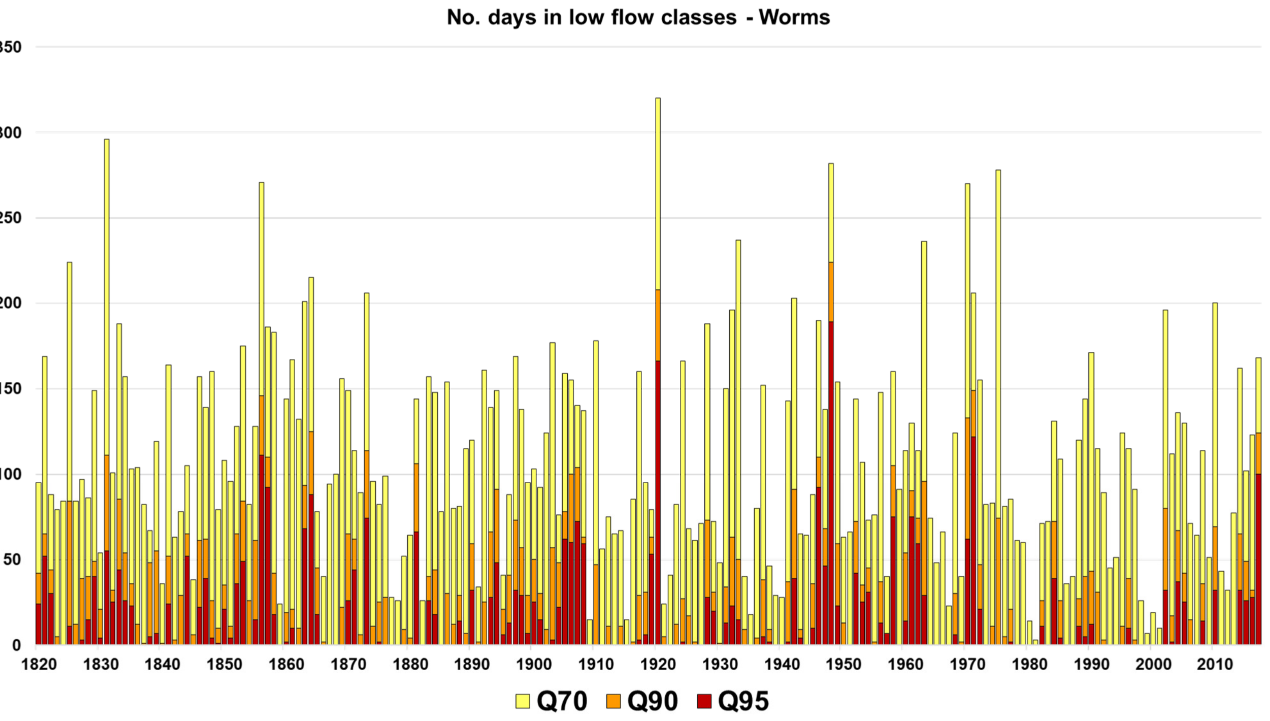

b)

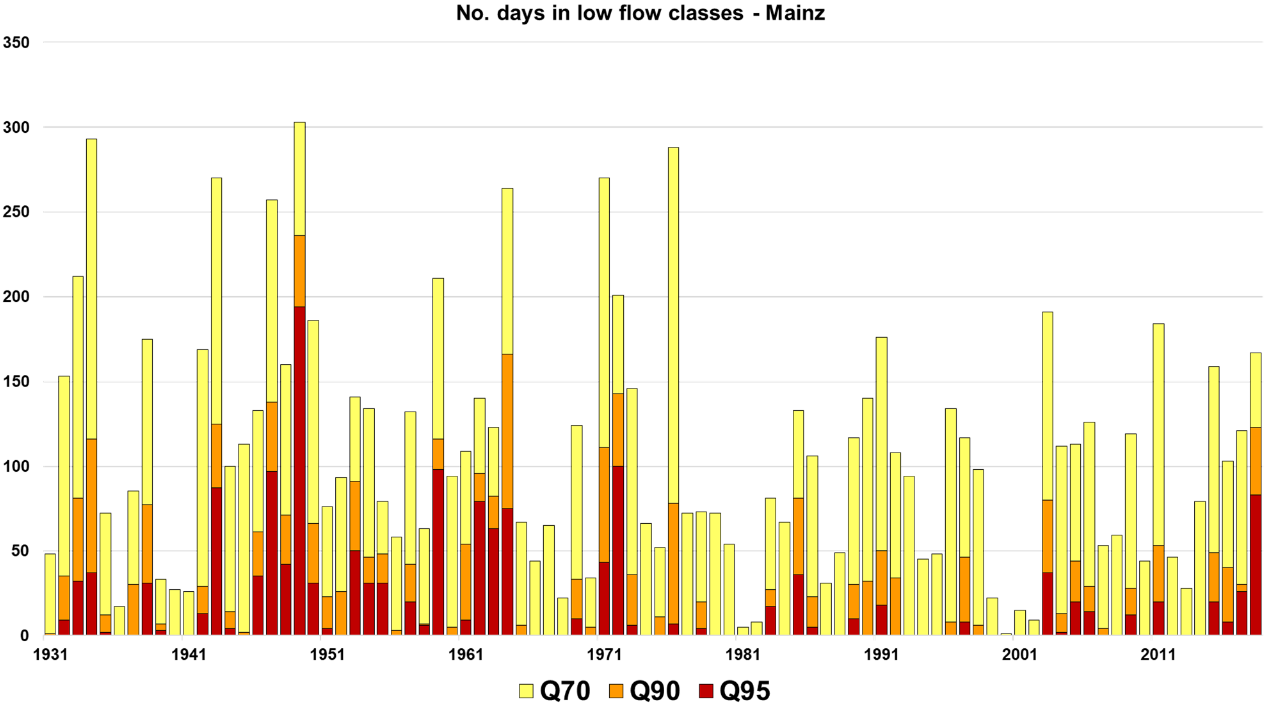

Figure S3. a) Number of days in low flow classes at Worms gauging station over the period 1820 – 2018 and b) Number of days in low flow classes at Mainz gauging station over the period 1931 – 2018

a)

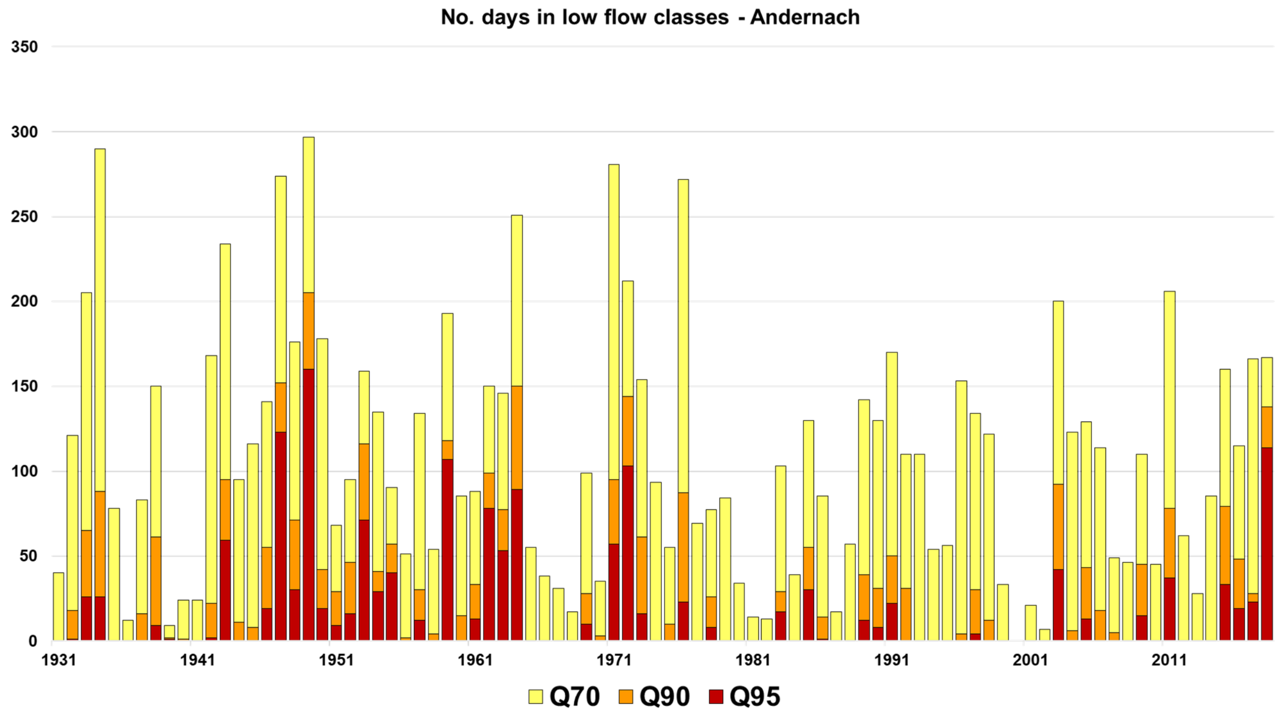

b)

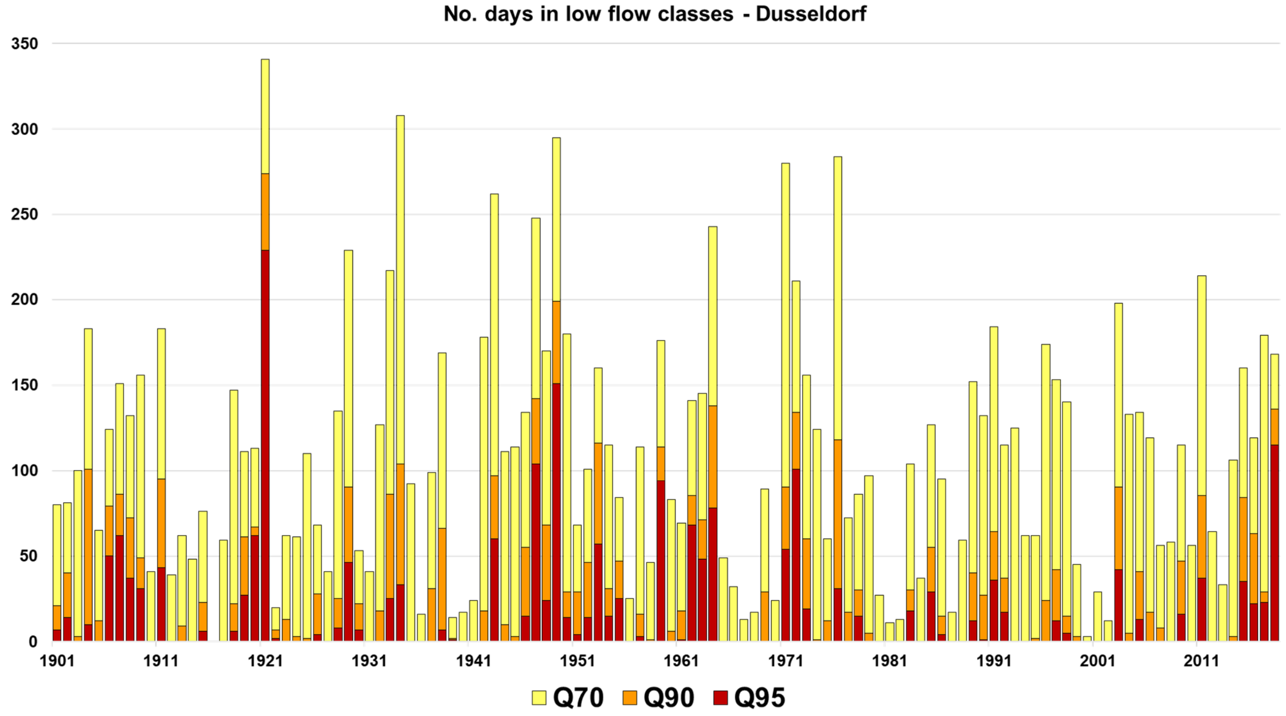

Figure S4. a) Number of days in low flow classes at Andernach gauging station over the period 1931 – 2018 and b) Number of days in low flow classes at Dusseldorf gauging station over the period 1901 – 2018

a)

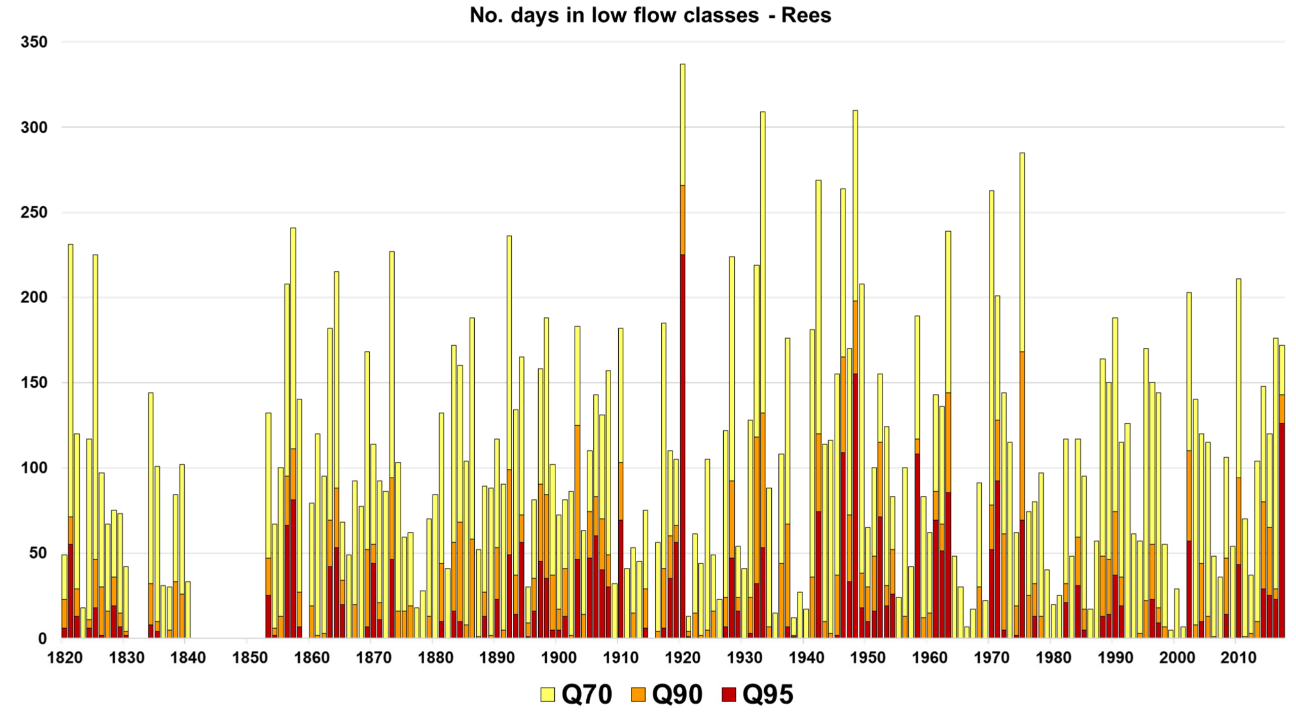

b)

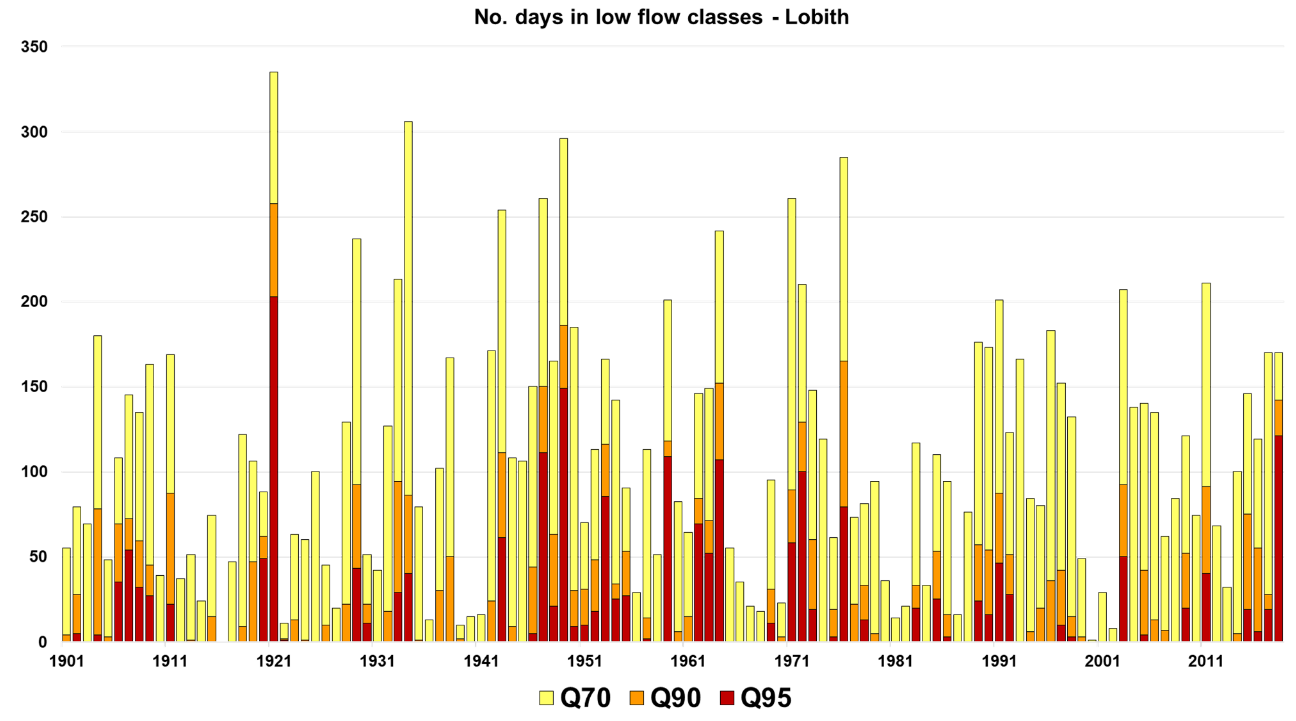

Figure S5. a) Number of days in low flow classes at Rees gauging station over the period 1820 – 2018 and b) Number of days in low flow classes at Lobith gauging station over the period 1901 – 2018

a)

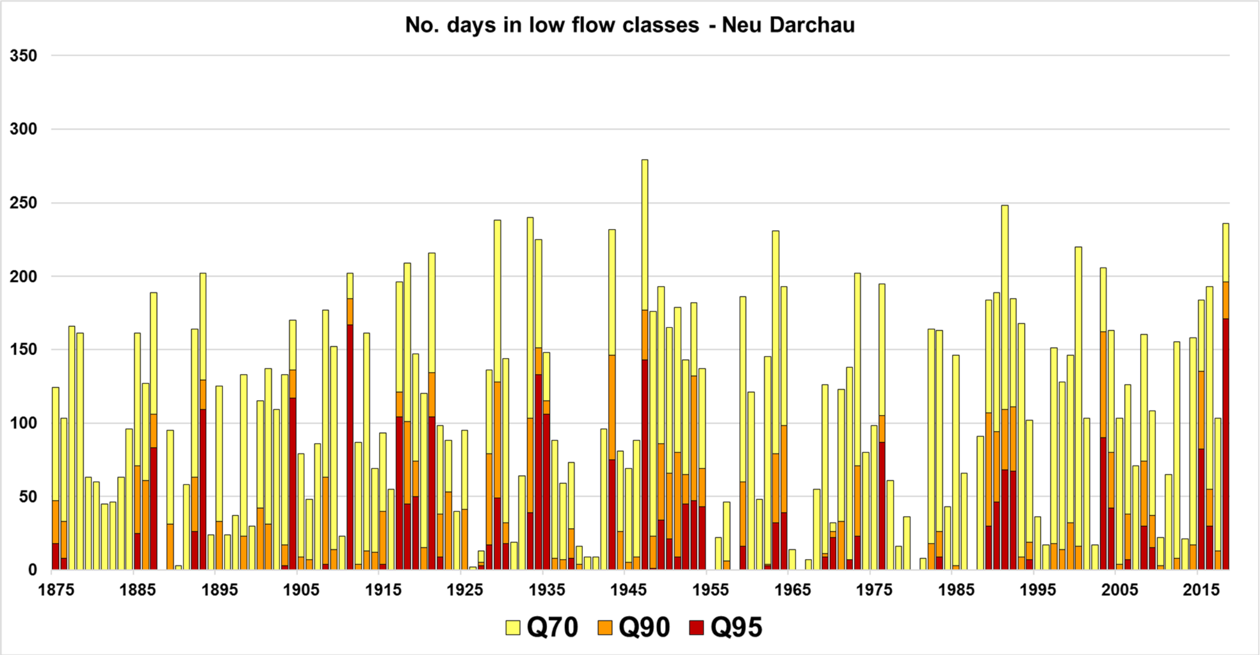

b)

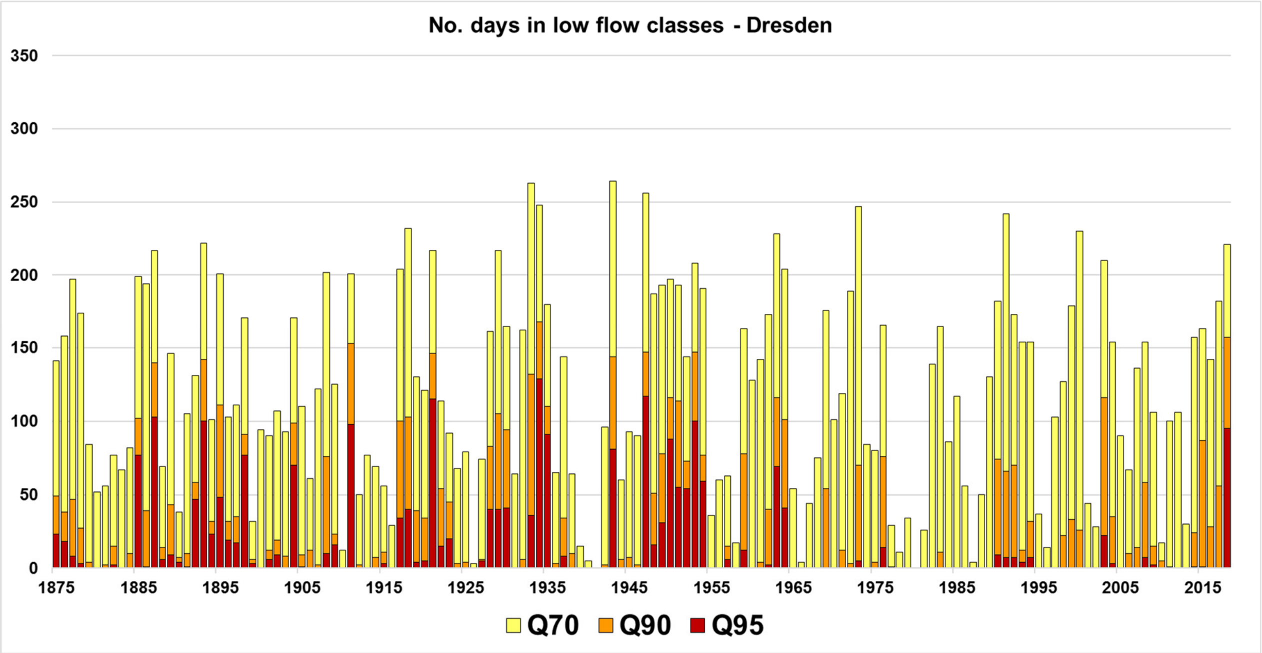

Figure S6. a) Number of days in low flow classes at Neu Darchau gauging station over the period 1875 – 2018 and b) Number of days in low flow classes at Dresden gauging station over the period 1875 – 2018.

a)

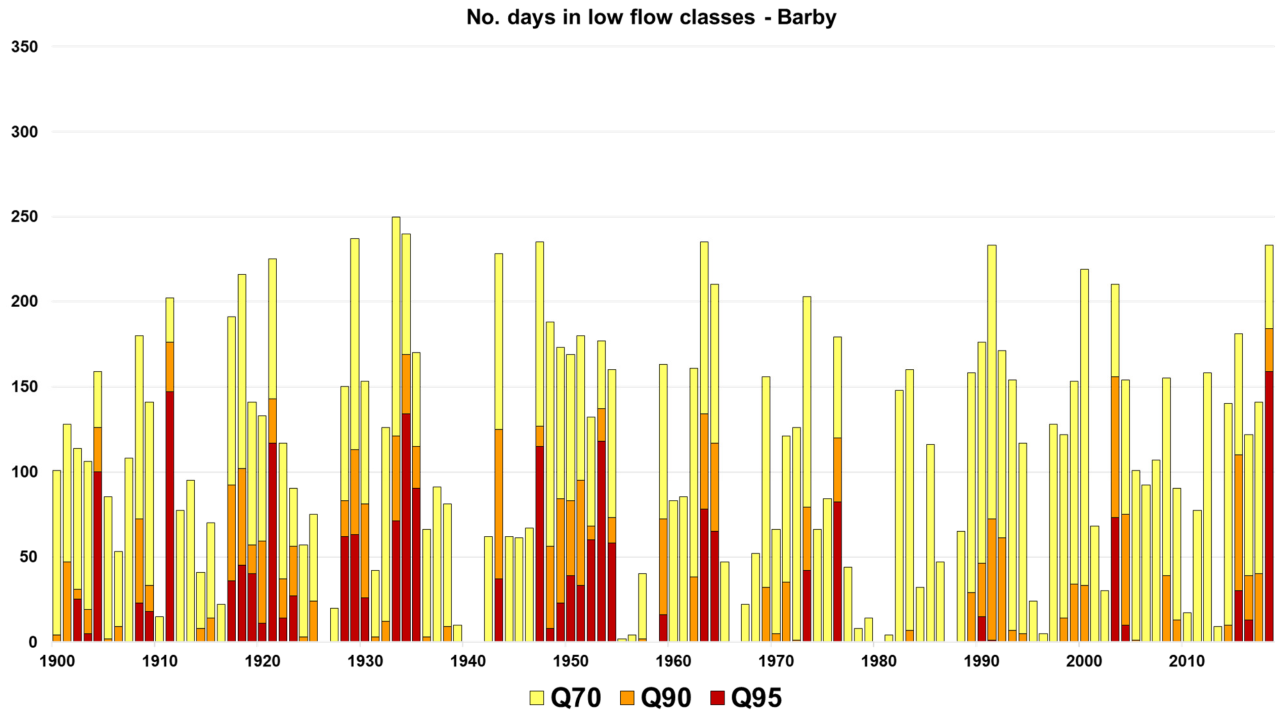

b)

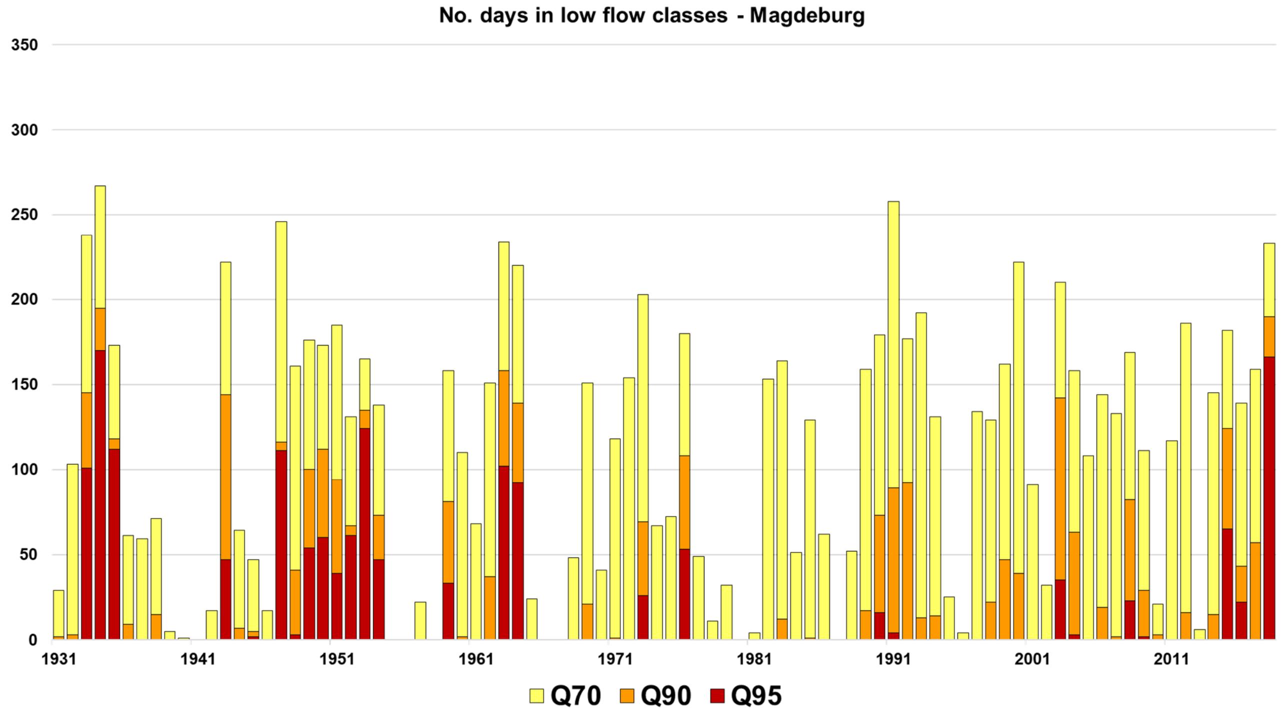

Figure S7. a) Number of days in low flow classes at Barby gauging station over the period 1900 – 2018 and b) Number of days in low flow classes at Magdeburg gauging station over the period 1931 – 2018

a)

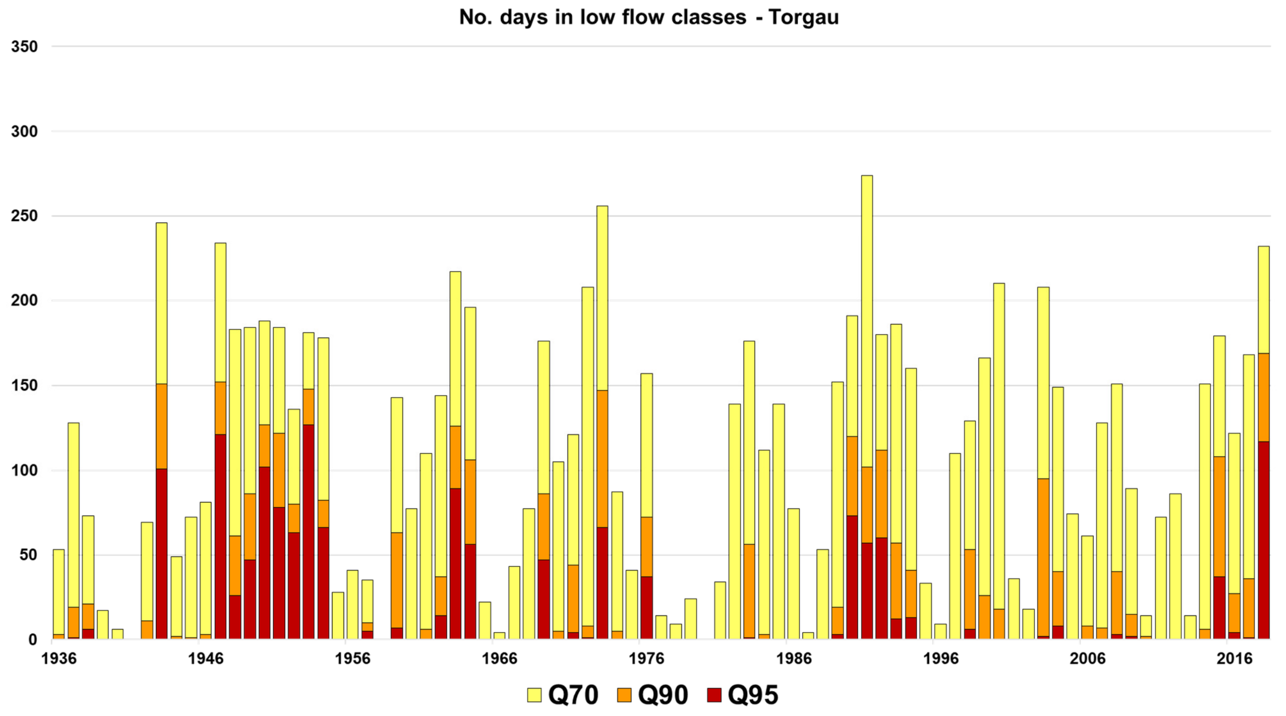

b)

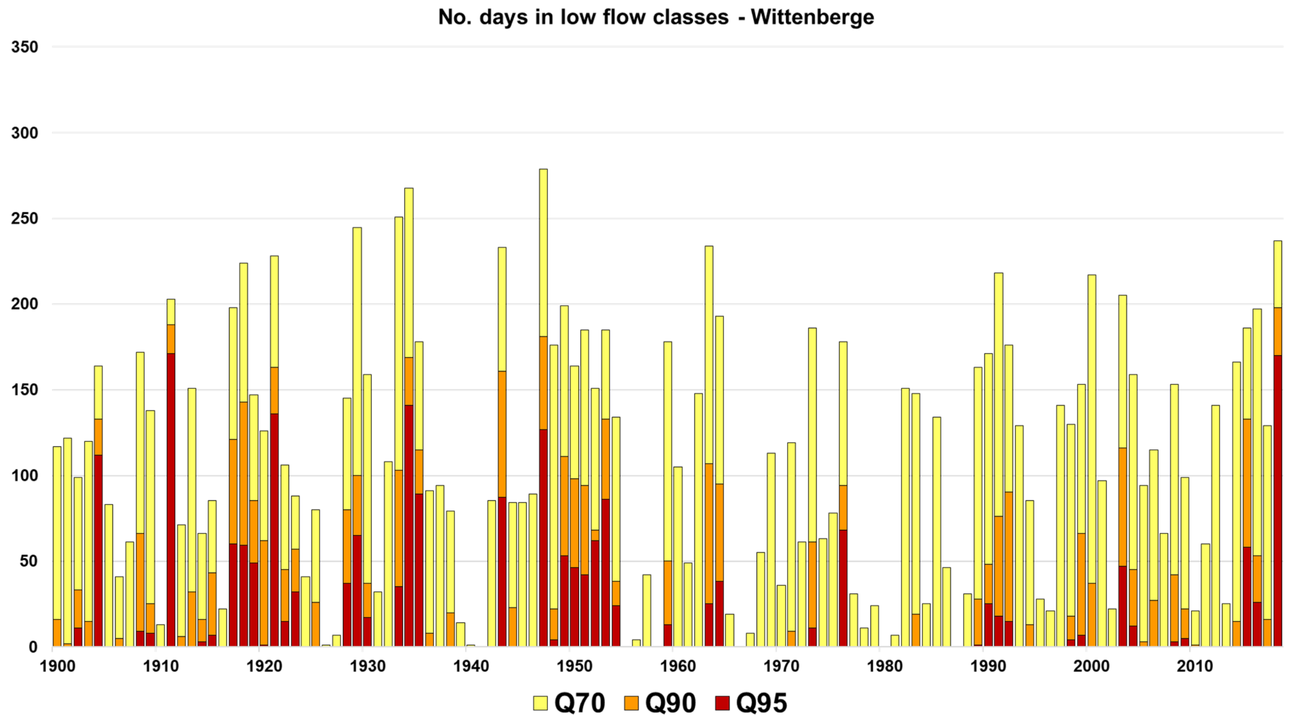

Figure S8. a) Number of days in low flow classes at Torgau gauging station over the period 1936 – 2018 and b) Number of days in low flow classes at Wittenberge gauging station over the period 1900 – 2018

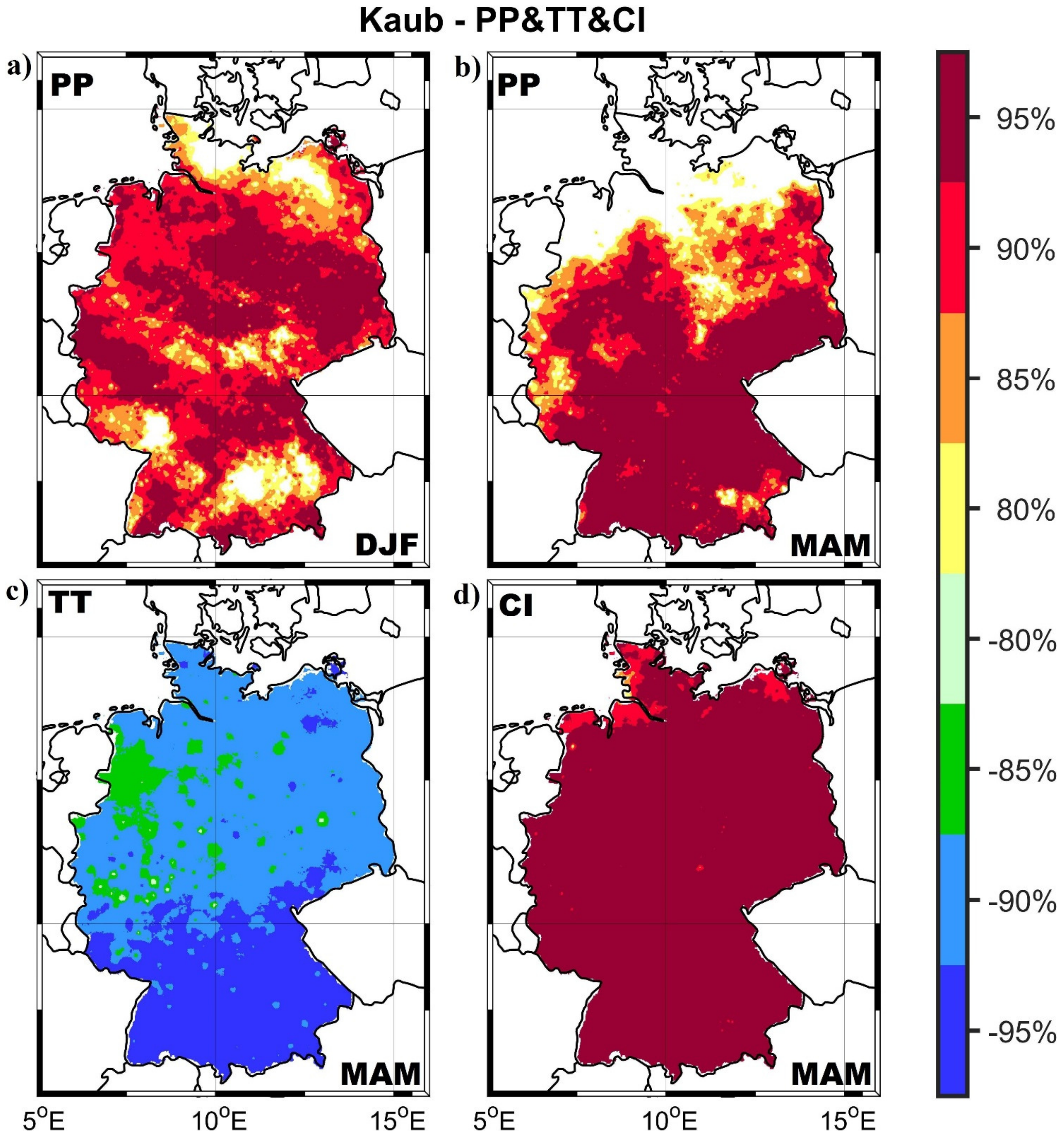

Figure S9. Stability map of the correlation between summer streamflow at Kaub gauging station and a) DJF PP, b) MAM PP, c) MAM TT and d) MAM CI. For PP, TT and CI all the regions where the correlation is stable and significant above the 90% significance level have been used in the forecast model.

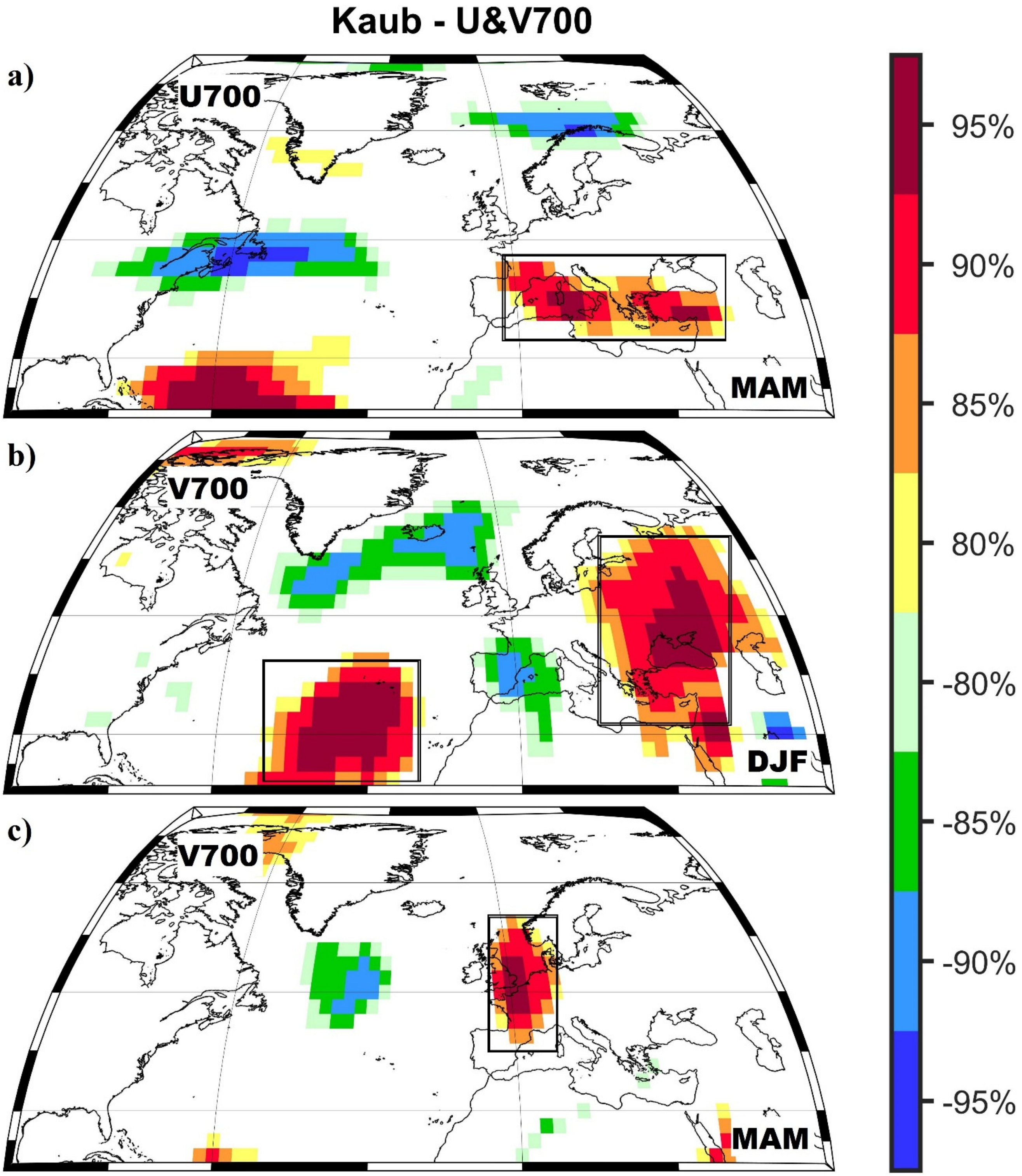

Figure S10. Stability map of the correlation between summer streamflow at Kaub gauging station and a) MAM U700; b) DJF V700 and c) MAM V700. The black boxes indicate the regions used for the summer streamflow at Kaub gauging station. Only the regions where the correlation was above 90% significance level were used in the forecast model.

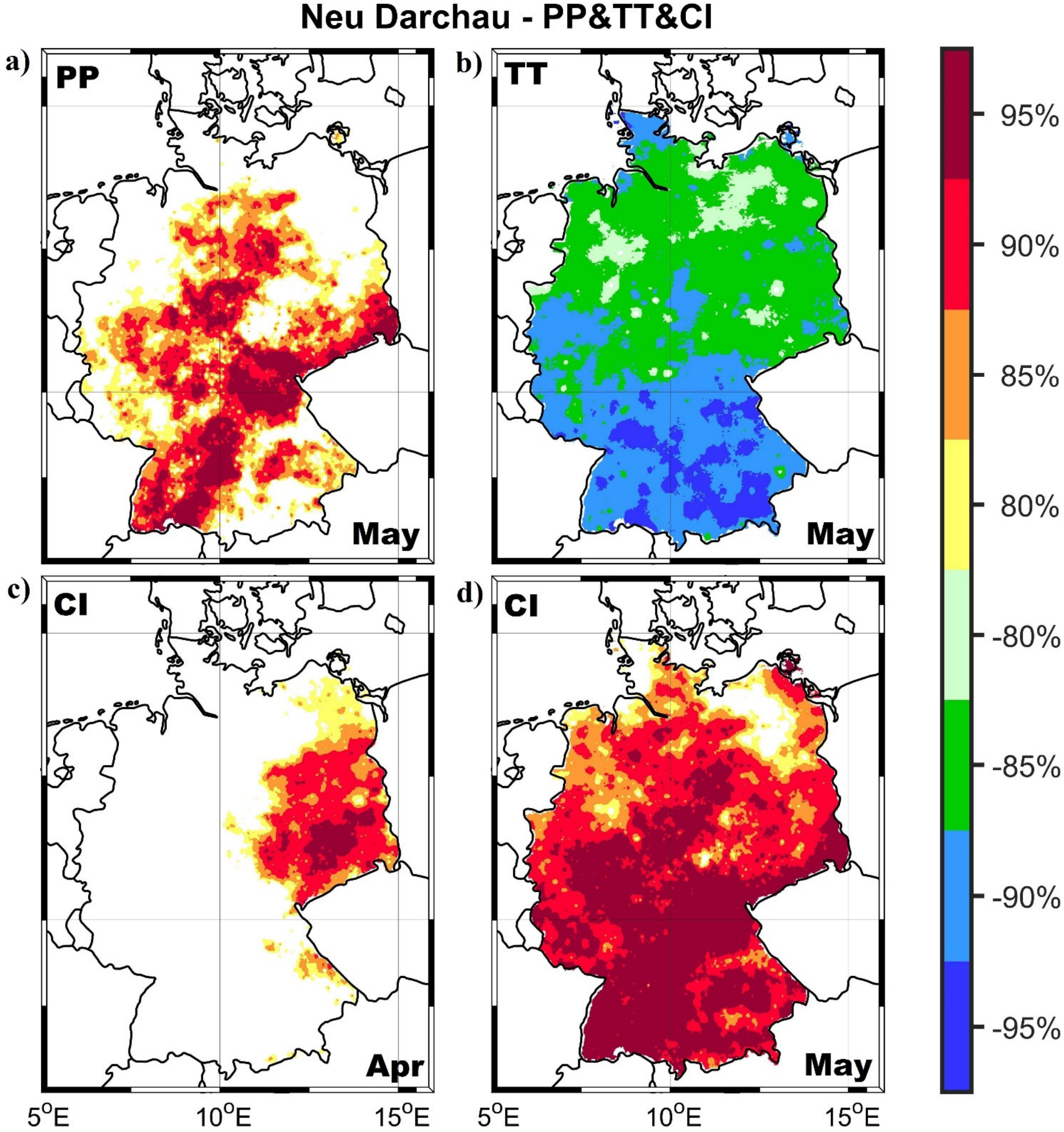

Figure S11. Stability map of the correlation between summer streamflow at Neu Darchau gauging station and a) May PP, b) May TT, c) April CI and d) May CI. For PP, TT and CI all the regions where the correlation is stable and significant above the 90% significance level have been used in the forecast model.

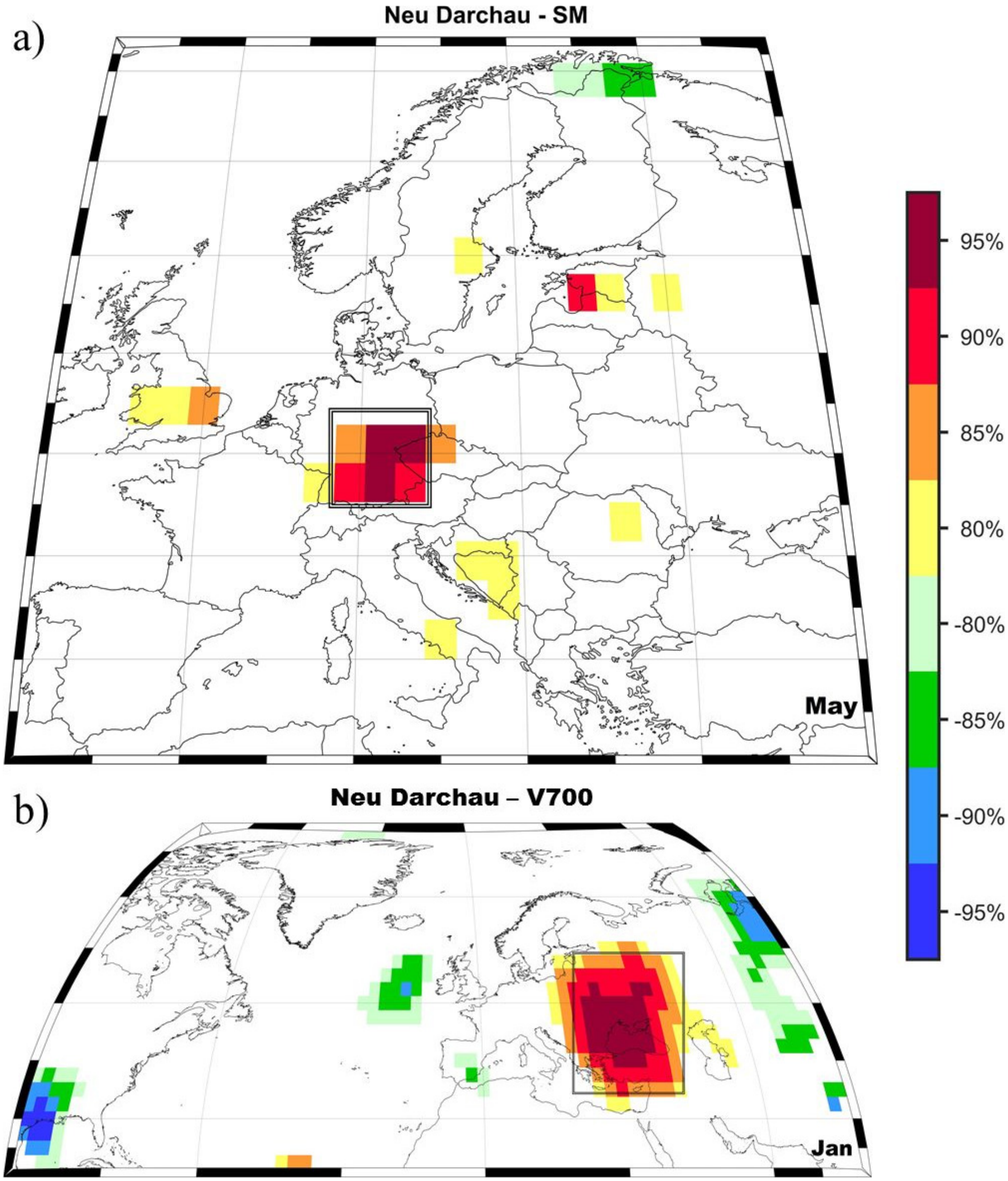

Figure S12. Stability map of the correlation between summer streamflow at Neu Darchau gauging station and a) May SM and b) January V700. The black boxes indicate the regions used for the summer streamflow at Neu Darchau gauging station. Only the regions where the correlation was above 90% significance level were used in the forecast model.

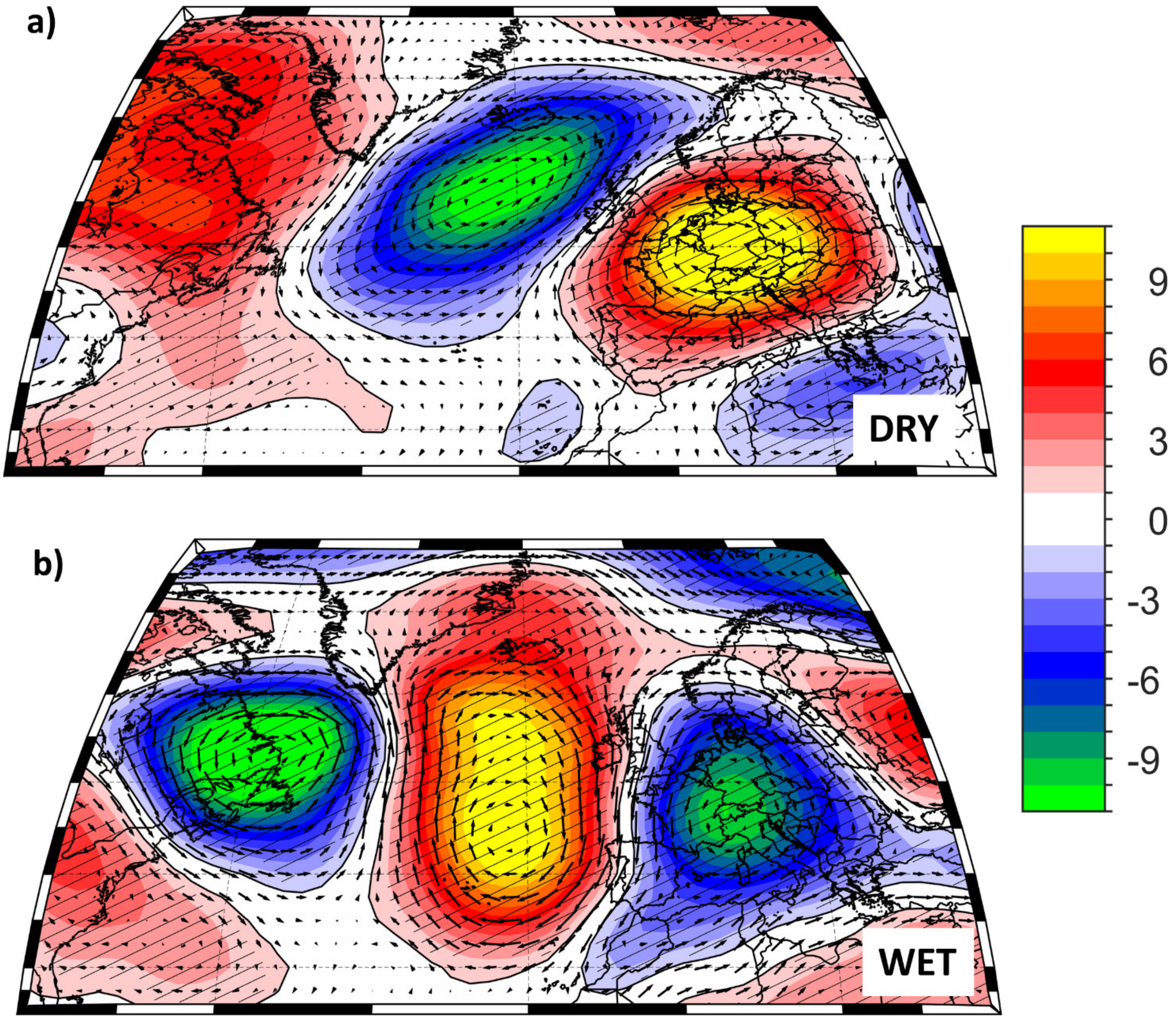

Figure S13. a) The composite map between low summer streamflow at Neu Darchau gauging station (< − 0.75 std. dev.) and summer Geopotential Height and wind vectors at 500mb (Z500) and b) The composite map between high summer streamflow at Neu Darchau (> 0.75 std. dev.) and summer Geopotential Height and wind direction at 500mb (Z500) . The hatching highlights significant values at a confidence level of 95 %. Analyzed period: 1948 – 2019. Units: Z500 (m).

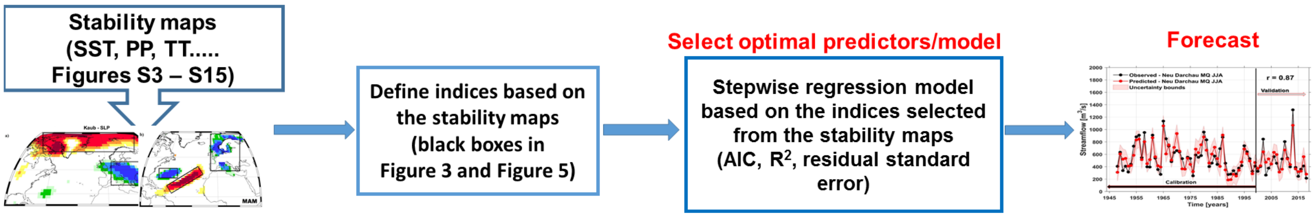

Figure S14. Workflow of the selection of the optimal predictors/model.

Table S1. Skill parameters (see paragraph 2 – skill measure for definition) based on different statistical methods for the observed and predicted summer streamflow at Kaub and at Neu Darchau gauging stations.

	Kaub		Neu Darchau	
	Calibration	Validation	Calibration	Validation
MAE	163.96	105.61	85.98	96.39
MSE	43359.21	15728.94	11537.04	15314.61
RMSE	208.23	125.42	107.41	123.75
NRMSE %	49.7	34.8	49.9	46.5
NSE	0.75	0.87	0.75	0.77
mNSE	0.49	0.63	0.51	0.47
rNSE	0.69	0.84	0.7	0.71
d	0.93	0.96	0.93	0.92
md	0.74	0.79	0.75	0.7
rd	0.91	0.95	0.92	0.9
r	0.87	0.94	0.87	0.9
R^2	0.75	0.89	0.75	0.81
KGE	0.83	0.8	0.85	0.69
